# Functional Landscape of Dysregulated MicroRNAs in Oral Squamous Cell Carcinoma: Clinical Implications

**DOI:** 10.3389/fonc.2020.00619

**Published:** 2020-05-12

**Authors:** Ruma Dey Ghosh, Arun Pattatheyil, Susanta Roychoudhury

**Affiliations:** ^1^Tata Translational Cancer Research Center, Tata Medical Center, Kolkata, India; ^2^Department of Head and Neck Surgical Oncology, Tata Medical Center, Kolkata, India; ^3^Saroj Gupta Cancer Centre and Research Institute, Kolkata, India

**Keywords:** dysregulated miRNA, miRNA biomarker, non-invasive biomarker, miRNA-based therapy, oral squamous cell carcinoma

## Abstract

MicroRNA (miRNA) dysregulation is associated with the pathogenesis of oral squamous cell carcinoma (OSCC), and its elucidation could potentially provide information on patient outcome. A growing body of translational research on miRNA biology is focusing on precision oncology, aiming to decode the miRNA regulatory network in the development and progression of cancer. Tissue-specific expression and stable presence in all body fluids are unique features of miRNAs, which could be potentially exploited in the clinical setting. Recent understanding of miRNA properties has led them to be useful, attractive, and potential tools either as biomarkers (distinct miRNA expression signature) for diagnosis and prognostic outcomes or as targets for novel therapeutic entities, enabling personalized treatment for OSCC. In this review, we discuss recent research on different aspects of alterations in miRNA profiles along with their clinical significance and strive to identify probable potential miRNA biomarkers for diagnosis and prognosis of OSCC. We also discuss the current understanding and scope of development of miRNA-based therapeutics against OSCC.

## Introduction

Oral squamous cell carcinoma (OSCC) is the second most common cancer reported in India. According to recent Globacon-2018 data, ~120,000 new cases of OSCC are detected every year in India (1, 2). It is the leading cause of cancer-associated death in the Indian male population. Annually, more than 72,000 deaths are attributed to this disease in this country (3). OSCC originates in mucosal epithelial cells of the oral cavity (4, 5). Tobacco (smoking/smokeless) and alcohol are the primary risk factors for OSCC. Chewing of areca nut, betel leaf, poor oral health hygiene, and human papillomavirus (HPV) infection are also important risk factors for OSCC. Treatment in the early stages of the disease offers the best chance of cure (4, 5). Surgery is the first line of treatment, whereas radiotherapy and chemotherapy are used as adjuvant therapies (6, 7). Treatment strategies mainly depend on the location of the primary tumor, identification of high-risk features by histopathology, stage of tumor, and comorbidities. The 5-year survival rate of patients with oral cancer is around 50%. Poor outcomes are attributed to disease recurrence (both second primaries and locoregional recurrence) and distant metastasis. Despite availability of risk information from histopathology, the pattern and timing of relapse and metastasis are difficult to predict. About 86% of the recurrence occurs within 24 months after primary treatment (7). Prognostic information is mainly derived from anatomic location; tumor stage; tumor thickness; and histological characteristics like cellular heterogeneity, degree of differentiation, depth of invasion, presence of nodal metastases, margin status, neural invasion, and pattern of invasion (6).

Biomarkers are quantifiable indicators associated with the specific disease conditions that facilitate decision-making by clinicians with respect to the most effective clinical interventions. In conventional practice, extra nodal extension (ENE), perineural invasion (PNI), and lymphovascular invasion (LVI) are considered histopathological biomarkers associated with poor disease prognosis (6, 7). Given that the genetics of OSCC is highly heterogeneous and complex, so far, no known molecular biomarker (except HPV positivity) has been used to subclassify OSCC accurately. Currently, only one epidermal growth factor receptor (EGFR)-specific targeted-immunotherapy with cetuximab antibody is available for the management of this cancer. Therefore, in the current scenario, early detection of the disease with identification of distinct prognostic subgroups to facilitate advanced treatment strategies is required for effective management of OSCC. Genomic, epigenomic, proteomic, and metabolomic high-throughput approaches have recently been used to discover and validate tumor biomarkers individually and/or in panels (8–13). Since microRNAs (miRNAs) are highly stable in tissues as well as in circulation, they are considered potential biomarkers for cancer detection and prognostication (14–18). Here, we review potential candidate miRNAs for their possible use as molecular biomarkers to improve the diagnosis and prognostication of OSCC and to stratify such patients into distinct prognostic subgroups. In this review, we have voyaged through various dysregulated miRNAs reported to be responsible for the pathogenesis, progression, and specific outcomes of OSCC. Their functional associations with overall therapeutic management, responsiveness, recurrence, and metastasis of OSCC are also elaborated upon.

## Basics of MicroRNAs: Altered miRNA Function can Fine-Tune Cell-Fate Decision via Altered Gene and Protein Expression

Mature miRNAs are endogenous, single-stranded, evolutionarily conserved, non-coding RNAs ~19–26 nucleotides long; discovered by Lee et al. (19). miRNAs preferentially interact with complementary seed sequences in the 3′ untranslated regions (UTRs) of their target mRNAs. miRNA binding sites may also be present in the 5′ UTRs and coding sequences of target mRNAs (20). miRNA-mediated gene silencing is a fundamental biological process for cellular homeostasis, executed through translational inhibition followed by mRNA deadenylation and decay (21–23). Circulating cell-free miRNAs also play an important role in intracellular/intercellular communication through miRNA-mediated gene silencing (17, 24). As a result, miRNA expression profiles in tissues and circulation are associated with several pathophysiological conditions, including cancers (17, 25). Dysregulation in miRNA expression profile was first reported in leukemia (26). Therefore, a distinct miRNA expression signature that distinguishes normal tissues from cancer tissues could be a new “hallmark of cancer,” which regulates almost all other cancer hallmarks defined earlier (18, 27, 28).

Functional dysregulation of mature miRNAs is associated with various kinds of pathological conditions. Dysregulation of miRNA expression and function may be due to one or more of the following: (1) altered miRNA biogenesis process: (a) epigenetic (methylation and histone modifications) alterations of miRNA genes, (b) altered activities of transcription factors, and (c) altered expression of miRNA-processing enzymes (Drosha, Dicer, etc.); (2) chromosomal instability, genomic instability, and presence of mutations in miRNA genes; (3) single-nucleotide polymorphisms (SNPs), deletions, and duplications in miRNA genes (pri-, pre-, and mature miRNA regions) as well as in the binding sequences on target mRNAs; (4) loss of miRNA-binding sites on target mRNAs; and (5) redirecting of the miRNA-induced silencing complex (miRISC) to multiple competitive miRNA-binding sites present in competing endogenous RNAs (ceRNAs), which act as miRNA sponges and inhibit their functions (17, 23, 28–30). All ceRNAs are naturally occurring, endogenous regulatory molecules, like long non-coding RNAs (lncRNAs), circular RNAs, pseudogenes, and some protein-coding mRNAs (29, 30). Notably, in miRNA-mediated regulatory networks, one miRNA can regulate many genes and a single gene can be regulated by many miRNAs (23).

The above-described processes ultimately lead to activation of some oncogenic genes/proteins and, at the same time, deactivation of some tumor suppressor genes/proteins. They also direct cellular signaling cascades toward making the cell fate verdict (23, 28). Eventually, these distinct alterations in miRNA expression profile drive a normal cell to transform into a cancer cell and dictate its progression, metastasis, stemness, and responsiveness to therapy (radiotherapy/chemotherapy) (17, 18).

## Distinct MicroRNA Signatures as Hallmarks of Oral Squamous Cell Carcinoma

The utility of miRNAs as diagnostic and prognostic biomarkers of OSCC has not been convincingly established. Previous studies on miRNA expression profiling mainly focused on differential expression of miRNAs in normal and tumor tissues and body fluids (blood, saliva, serum, or plasma) from OSCC patients (10, 12). These studies have yielded a plethora of dysregulated (upregulated/downregulated) miRNAs in OSCC. Here, we used the miRCancer database, PubMed, and Google to search for relevant data and reevaluate miRNAs as potential biomarkers for the diagnosis, prognosis, and therapeutics of OSCC. miRCancer is an online, up-to-date database (last updated on August 27, 2019) that provides a comprehensive collection of miRNA expression profiles in various human cancers extracted from published literature available in PubMed (31).

## MicroRNA Profiling in Oral Squamous Cell Carcinoma

### Dysregulated miRNA Expression in Tumor Tissues

Tran et al. (32) performed the first high-throughput miRNA profiling in OSCC using nine OSCC cell lines. Subsequently, several investigators have studied miRNA profiles as prognostic biomarkers using primary tumor and paired control tissues from patients with head and neck squamous cell carcinoma (HNSCC) (33–35). The differential expression analysis revealed sets of mature miRNAs that were either upregulated or downregulated in the tumor tissues. Childs et al. (35) showed that miR-21, miR-155, miR-191, and miR-221 were upregulated, whereas miR-1, miR-133a, miR-205, and let-7d were downregulated in primary tumors at diagnosis (35, 36). Another retrospective study on 51 formalin-fixed HNSCC tumor samples revealed consistent expression of mature miRNAs in malignant tissues; miR-21, miR-155, let-7i, miR-142-3p, miR-423, miR-106b, miR-20a, and miR-16 were overexpressed, whereas miR-125b, miR-375, and miR-10a were underexpressed) (37). Lu et al. (38) showed that miR-10b, miR-196a, miR-196b, miR-582-5p, miR-15b, miR-301, miR-148b, and miR-128a were upregulated, whereas miR-503 and miR-31 were downregulated in six oral cancer cell lines compared with those in normal keratinocytes. Specific miRNAs related to the clinicopathological features of site-specific OSCC were investigated, which demonstrated a significant difference in let-7a, miR-200c, miR-34a levels between oropharyngeal and laryngeal cancers (39). In addition, miR-21, miR-200c, and miR-34a were upregulated, and miR-375 was downregulated in tumor tissues of all subsites when compared with those in paired control tissues (39). Studies also suggested that genes associated with the phosphoinositide 3-kinase (PI3K)/AKT and p53 signaling pathways, which are involved in the OSCC carcinogenesis process, were regulated through a set of dysregulated miRNAs, such as let-7a, let-7d, let-7f, miR-16, miR-29b, miR-142-3p, miR-144, miR-203, and miR-223 (40). Lamperska et al. (41) suggested that miR-21 and miR-205 could be used to analyze the clarity of surgical margins, but they failed to find a correlation between miRNA expression and clinical outcome and the course of illness. Here, we considered only those studies that reported targeted or genome-wide miRNA profiling either in cell lines or in tumor samples.

These studies make it obvious that malignant OSCC tumors have distinct miRNA expression profiles. Subsequently, the regulatory network composed of these distinct miRNAs in the malignant cell causes key downstream molecular alteration, which ultimately leads to a distinct patient outcome (17, 28, 42). It is evident from the above studies that the results are not always consistent. The source of the problem could be found within and among the studies. Variations are observed in study design, end point objective, selection of cell lines, use of appropriate controls (in most of the cases, adjacent normal tissues were used which could harbor genomic alterations), methodology, protocol or treatment strategies, and localized patient pools in these studies. Further, purity and availability of tumor tissue, stromal cell contamination, and small sample size may impact study results. In most of the studies, intra/inter tumor heterogeneity, variable etiopathogenesis, and heterogenous genetic constitution of each patient are important factors that lead to variations in results. Despite these limitations, the results of individual studies demonstrate that miRNAs may be useful as potential biomarkers to predict OSCC outcome. The present quantum of knowledge lays the groundwork for logical implementation and execution of large-scale studies with improved, standardized study protocols in the future.

### miRNA Expression Signatures Associated With Risk Factors of OSCC

One miRNA profiling study comparing smoker and non-smoker patients reported high miR-155 expression in 58% of OSCC cases and 83% of dysplasia cases and subsequently suggested miR-155 as a driver of oral tumorigenesis in non-smokers (43).

The association of OSCC with betel quid was also analyzed in OSCC specimens by another investigator who discovered 84 betel quid-associated mature miRNAs, of which 19 were located on chromosome 14q32.2 (44). In this context, Hou et al. (45) established that specific polymorphisms in miR-499a are associated with OSCC progression. They discovered that the T/C+C/C genotypes of miR-499a increased the risk of betel quid-associated oral submucosal fibrosis (OSF) but decreased the risk of OSCC. Further, miR-499a T>C (rs3746444) influences the expression of miR-499-5p during OSCC carcinogenesis (45).

Several investigators have also conducted miRNA expression profiling to compare HPV-positive and -negative OSCC tumors and revealed that miR-127-3p, miR-363, miR-20a, miR-34a, let-7c-5p, and miR-9 could effectively distinguish between the two groups (39, 46–49). Different etiological factors causing distinct patterns of miRNA expression have also been well-established by several investigators. Most of the work has been done on HPV-associated OSCC. The studies reveal little effect of tobacco and betel quid on miRNA expression in OSCC compared with that in HPV-associated OSCC.

### Dysregulated miRNAs in Tumor Cells Collected Through Non-invasive Brush Biopsy

Recently, the more advanced brush biopsy or other related scraper-based methods have offered non-invasive ways to identify OSCC-specific miRNA biomarkers for diagnosis and prognostication of the disease. Gissi et al., using brush biopsy samples from OSCCs and from regenerative areas after surgical resection and from their respective normal distant mucosa, revealed that miR-146a and miR-191 were significantly altered in the regenerative areas after OSCC resection (50). Studies also claim that brush biopsy samples may be superior to surgically dissected samples (51, 52). In brush biopsy, sampling sites within lesions that are not ulcerated and are non-necrotic and minimally friable make the samples homogeneous, with viable epithelial cells. It must be noted, however, that in case of smaller tumors (T1, T2), there is a chance of normal epithelial cell contamination in brush biopsy samples (51, 52). For high-throughput technology (next-generation sequencing), obtaining high quality and quantity of total RNA (or enriched miRNA) from brush biopsy samples is the main challenge (51). Quantitative real-time PCR (qRT-PCT) is a reliable method in this case. miRNA profiles in individual brush biopsy OSCC samples show ~50% overlap with miRNAs enriched in surgically obtained tumor tissue profiles (51). The non-invasive rapid brush biopsy methods are useful in obtaining homogeneous tumor cells. Over the years, given all the limitations, the accuracy in predicting OSCC-associated miRNA expression signature is still to be improved for clinical applications.

## Cell-Free MicroRNA Profiling: Potential Biomarkers for Liquid Biopsy

The most fascinating aspect of miRNA biology is the stable presence of cell-free miRNAs in all biological fluids. Previous studies have demonstrated that the stability of circulating cell-free miRNA results from either internalization of miRNAs into exosomes or other microvesicles or formation of complexes between circulating miRNAs and specific proteins and lipids (14–16, 53). These cell-free miRNAs are probably released by cancer cells, necrotic cells, and/or apoptotic cells along with their associated proteins or lipids, such as the RNA-binding protein NPM1 (nucleophosmin), AGO proteins (argonaute 1/2), and high-density lipoprotein (HDL), to avoid RNase degradation in blood circulation (15, 16, 54). Further, the spectrum of these cell-free miRNAs is altered by various pathophysiological conditions, including cancer (42, 54–57). In OSCC, saliva is one of the important sources to identify reliable biomarkers for predicting diagnosis and prognosis. Blood (serum and plasma) is another important sampling source. The sources of these cell-free miRNAs are thought to be directly associated with tumor pathogenesis and/or other related systemic physiological (immune system/metabolic system) conditions (54–56).

### Dysregulated Cell-Free miRNAs in Biological Fluids of OSCC Patients

#### Saliva

Studies using human saliva samples have shown that salivary cell-free miRNAs could be potential diagnostic biomarkers in OSCC patients compared with those in healthy individuals (58–60). Genome-wide expression patterns of miRNAs have revealed that miRNA expression is significantly altered in the saliva of OSCC patients compared with that in healthy controls. miR-125a, miR-136, miR-147, miR-1250, miR-148a, miR-200a, miR-632, miR-646, miR-668, miR-877, miR-503, miR-220a, and miR-323-5p were downregulated, and miR-24 and miR-27b were found to be upregulated. The studies revealed that miR-27b was significantly upregulated in OSCC patients compared with that in healthy controls, patients with OSCC in remission, and patients with oral lichen planus and served as a biomarker to detect OSCC. Finally, the studies concluded that miR-27b could be a valuable cell-free biomarker in saliva for distinguishing OSCC patients (60).

#### Plasma

A study using plasma samples obtained at different time points showed that plasma miR-146a levels were significantly higher in OSCC patients (sensitivity: >0.72) than in healthy controls, and these levels decreased drastically after tumor resection in these patients (61). Similarly, higher plasma miR-187-3p level was found to be another potential marker of OSCC diagnosis, and the plasma levels of miR-187-3p were significantly reduced after tumor resection in patients who had better prognosis (62). Studies have also suggested that circulating miR-196a, miR-196b, and miR-200b-3p levels in plasma might serve as a panel of plasma biomarkers for the early detection of oral cancer (63–65). In a separate study, three plasma miRNAs—miR-222-3p, miR-150-5p, and miR-423-5p—were identified for early detection of malignant OSCC (66). miR-222-3p and miR-423-5p negatively correlated with T stage, lymph node metastasis status, and clinical stage. A high diagnostic accuracy (area under curve = 0.88) was demonstrated for discriminating oral leukoplakia from OSCC (66).

#### Serum

A miRNA microarray experiment using serum samples from OSCC patients vs. healthy controls revealed 16 miRNAs were significantly upregulated and 10 were significantly downregulated in the patients. miR-483-5p expression was significantly correlated (sensitivity = 0.853, specificity = 0.746) with lymph node metastasis and shorter survival, suggesting increased miR-483-5p expression in OSCC and suggesting its potential as a novel diagnostic and prognostic biomarker for OSCC (67). Further, low serum miR-9 level correlated with poor prognosis of OSCC (68). Recently, another study compared the circulating miRNA profile with the respective tumor-specific dysregulated miRNA profile and suggested that hsa-miR-32-5p in serum is a potential non-invasive biomarker for OSCC (69).

#### Whole Blood

Circulating miR-21 level and *PTEN* expression observed in whole blood samples could be possible biomarkers for detection of OSCC (70). Ries et al. (71–73) suggested that whole-blood sample is more reliable than only one specific blood component (serum/plasma/circulating cells) for identifying miRNA biomarkers for OSCC using a minimally invasive method. Microarray-based miRNA expression profiling was performed on 20 whole-blood samples (in a PAXgene blood RNA tube) from OSCC patients and healthy volunteers, and the results were validated through qRT-PCR using another 57 OSCC patient samples and 33 healthy control samples. This study showed that miR-186 was downregulated and miR-3651 and miR-494 were upregulated significantly in OSCC (71). In further studies, the authors evaluated these circulating miRNA biomarkers with diagnostic and prognostic significance in different patient cohorts (72). They also showed that the circulating miRNA expression signature (from whole-blood sample) was different from the miRNA expression in OSCC tissues (73). This is probably because the changes in miRNA expression in circulation occur as a consequence of pathogenic reactions upon immune-pathogenic interactions in response to cancer.

On the basis of all these studies, we can suggest that liquid biopsy would be a reliable, consistent, rapid, easy, cheap, and minimally invasive method to determine miRNA expression signatures to predict OSCC diagnosis and prognosis (57, 74). Evident challenges persist in terms of quality and quantity (for high-throughput techniques) of RNA and usage of proper endogenous controls for data normalization (75–77). To overcome these issues, recently advanced instruments (Qubit, concentrator, droplet digital PCR) and advanced modified assay protocols (inclusion of exogenous spike-in-control and newly identified endogenous cell-free control miRNA) have been introduced to obtain reliable, potential predictive biomarkers for OSCC (75, 77, 78). We prepared a list of dysregulated miRNAs, in which each miRNA is representative of a particular miRNA expression signature in tumor tissues/cell lines and/or in circulation/other body fluids (Supplementary Table 1). Individual studies on the effect of one/two miRNAs with clinical significance were also included in this table. Each miRNA is accountable either for sole or cumulative functions related to OSCC pathogenesis, progression, differential tumor behavior, aggressiveness, invasion, and metastasis, resulting in distinct outcome for each patient with OSCC.

## Clinical Significance of Dysregulated MicroRNAs in the Diagnosis and Prognosis of OSCC

### miRNA Signature for Susceptibility to OSCC

A major goal of precision medicine is to assess disease risk based on the genetic makeup of an individual. SNPs in various miRNAs have also been shown to be associated with different cancers. Dysregulation due to distinct polymorphisms in mature miRNAs, particularly miR-196a2 rs11614913 C>T, miR-146a rs2910164 G>C, miR-149 rs2292832 C>T, and miR-499 rs3746444 A>G, are associated with the risk of OSCC (45, 63, 79). In addition, polymorphisms in miR-146a [genotype: CC vs. GG + CG; odds ratio (OR) = 0.874, *p* = 0.041] and miR-196a2 (genotype: TT vs. TC + CC; OR <1, *p* < 0.05) increase the risk of OSCC, whereas the miR-499 polymorphisms (G allele and the GG genotype; OR > 1, *p* < 0.05) exert protective effects against OSCC risk. In this context, study results on miR-149 polymorphisms are not significant. They are associated with both increased risk of nodal metastasis and poor survival in OSCC, although another research group disclosed that they appeared to have no significant relationship with the risk of OSCC (80, 81).

### miRNAs as Early Biomarkers for OSCC Diagnosis

Early detection of OSCC allows clinicians to provide proper administration of curative treatment long before it metastasizes and progresses to the advanced stages. The identification of biomarkers for early detection and prognostication of OSCC through minimally invasive or non-invasive methods acquires major emphasis in current investigative drives. A targeted miRNA expression profiling study (using 22 oral leukoplakia tissue samples with different grades of dysplasia, 17 OSCC samples, and six normal oral mucosa samples) demonstrated the prognostic values of miR-21, miR-181b, and miR-345 in oral leukoplakia with severe dysplasia. Although dysplasia grading is not a very reliable predictor, advanced aggressive dysplasia progresses to OSCC (82). Other studies using tumor tissues revealed that miR-137 and miR-29a/b/c could be potential biomarkers for early diagnosis of OSCC. miR-29s (miR-29a/b/c) were significantly downregulated in OSCC patients (83). Consecutively, circulating miR-223 and miR-10b in plasma were proposed to be potential biomarkers for early detection of oral cancer (38, 84). Further, miR-146a, miR-187-3p, miR-196a, miR-196b, miR-200b-3p, miR-222-3p, miR-223, miR-150-5p, and miR-423-5p levels in plasma could also be potential diagnostic markers for early detection of OSCC (61–66). A separate study described serum miR-32-5p as a potential biomarker for OSCC (69).

### miRNAs as Biomarkers for OSCC Prognostication

#### Lymph Node Invasion and Distant Metastasis in OSCC

Epithelial–mesenchymal transition (EMT) of cancer cells is directly associated with cellular migration/invasion leading to cancer metastasis. In OSCC, ample dysregulated miRNAs have been found to be involved in EMT, invasion, and metastasis, which are primarily responsible for lymph node metastasis and distant metastasis.

In preclinical OSCC cell lines, miR-130b, miR-134, miR-149, miR-181d, miR-146b, miR-491, and miR-27a-3p are associated with EMT and cellular migration through targeting BMI1, MMP9, E-cadherin, and the YAP1-OCT4-Sox2 signaling axis (85, 86). High levels of miR-1275 and low levels of miR-222-3p and miR-423-5p are correlated with induced regional lymph node invasion in OSCC (40). Similarly, other studies have suggested that upregulation of miR-187, miR-196b, miR-372, miR-373, and miR-483 could be potential biomarkers for nodal metastasis in HNSCC (38, 62, 67, 87–90). ZEB1, Twist, and Snail (EMT-related transcription factors) are directly regulated by miR-429 and miR-101 and inversely by let-7d and mediate tumor growth and metastasis in OSCC (35, 91–93). Downregulation of miR-300 is another requirement for EMT initiation and maintenance, mediated through modulation of Twist expression and the transforming growth factor (TGF)β signaling pathway in OSCC (94). Bufalino et al. (95) demonstrated that lymph node metastasis resulted from downregulation of the miR-143/miR-145 cluster and consequent induction of activin-A, which contributed to poor prognosis through induced EMT. Similarly, RUNX2 is directly regulated by miR-376c-3p, which was found to be downregulated and to promote lymph node metastasis in OSCC (96). Other studies also demonstrated that the miR-23b/27b cluster regulates the MET oncogene, whereas miR-29a/b/c regulates the expression of *MMP2, LAMC2*, and *ITGA6*, responsible for disease invasion and metastasis in OSCC (83, 97, 98). Further, miR-218 is directly correlated with increased invasion and cellular migration mediated through *LAMB3* and *RICTOR* and the focal adhesion and mTOR-Akt signaling pathways (99, 100). *EGFR, c-MET*, and *KRAS* are direct targets of miR-1 and miR-206. Both these miRNAs, particularly miR-206, are significantly associated with advanced tumor node metastasis (node positivity in the Tumor Node Metastasis staging system) and shorter overall survival in OSCC (101, 102). Moreover, high expression of miR-196a and miR-149 polymorphisms is associated with increased risk of nodal metastasis (63, 80).

#### Biomarkers for Locoregional Recurrence in OSCC

The evolution of second primary or locoregional recurrence is unpredictable. In most cases, relapses are detected in late stages, which significantly reduce survival and worsen morbidity. Salvage surgery can cure recurrent tumors effectively if detected early.

Low miR-422a expression in stage III–IV tumors promotes local recurrence *via* targeting oncogenic *CD73* when compared with that in oropharynx stage III–IV tumors, without relapse or with locoregional relapse within 2 years of posttreatment (103). Other studies have shown that miR-196a, miR-205, and miR-675 are significantly associated with locoregional recurrence at diagnosis and treatment in OSCC (35, 80, 103, 104). miR-451 was found to be significantly overexpressed (4.7-fold) in non-relapsed vs. relapsed patients (37). Furthermore, locoregional recurrence in OSCC is also significantly affected by polymorphisms in miR-196a (63). All these indicators could be building blocks for developing meaningful biomarkers for early disease prognostication, recurrence, and/or metastasis in the clinical setting.

#### Dysregulated miRNAs Associated With Response to Chemoradiation Therapy in OSCC

Until date, cetuximab is the only clinically applied targeted drug used to treat patients with OSCC. However, the occurrence of therapeutic resistance or non-responsiveness has been found during chemotherapy/radiotherapy treatment in patients with OSCC. Several studies identified miRNAs as potential biomarkers to predict the sensitivity/resistance of tumors to chemotherapy or a particular drug used in chemotherapy and radiotherapy for OSCC.

Henson et al. (105) showed that the amplification of chromosomal band 11q13, loss of distal 11q, and downregulation of miR-125b and miR-100 are associated with radioresistance and disease progression (105, 106). In our previous study, we identified six cisplatin resistance-specific signature miRNAs—miR-130b, miR-134, miR-149, miR-181d, miR-146b, and miR-491. These miRNAs function in OSCC mainly through modulating the expression of proteins related to cancer stem cells (augmentation of CD44, c-Myc, and Oct-4), drug resistance (upregulation of P-gp and MRP1), and EMT (increase in BMI1 and MMP9 expression and loss of E-cadherin) (85). Low miR-29a level is reported to be associated with induced drug resistance and invasion (97). High miR-196a and miR-21 levels enhance radioresistance through inhibiting annexin A1 and signal transducer and activator of transcription 3 (STAT3), respectively (107, 108). Moreover, low Dicer expression is associated with resistance to 5-fluorouracil-based chemoradiotherapy and shorter overall survival in patients with OSCC (109). In brief, upregulation of the let-7 family, miR-203, miR-23a, miR-214, miR-518c, and miR-608 and downregulation of miR-21 and miR-342 have been shown to be connected with the manifestation of chemosensitivity/chemoresistance in OSCC (91, 110, 111). Moreover, therapeutic resistance is mediated through EGFR and c-MET, which are further alleviated by low levels of miR-1 and miR-206 in OSCC (102). However, determination of the exact range (single/panel) of these miRNA biomarkers as well as the spectrum of their expression level needs to be extremely accurate, sensitive, and specific in order to predict optimum therapy response in OSCC.

#### Biomarkers for Prediction of Patient Survival in OSCC

The relation between disease-free survival and overall survival of OSCC patients and aberrant miRNA expression has been studied by several investigators. Early detection and prompt treatment using suitable multidisciplinary protocols could improve survival in OSCC. Earlier evidence has shown that irrespective of tumor size, poor patient survival is significantly correlated with lower expression levels of miR-9, miR-149, miR-150-5p, miR-200b, miR-205, miR-375, miR-483-5p, miR-542-3p, and let-7d (35, 62, 68, 103, 104, 112–118). Concurrently, it was also found that overexpression of miR-1246 and miR-675 and downregulation of miR-187 and miR-134 in plasma are associated with better patient survival in OSCC (35, 62, 67, 68, 103, 104, 112–118). Moreover, other studies discovered that decreased levels of Dicer and miR-206 correlate significantly with lower overall survival in OSCC (101, 109).

## Advanced Therapeutics Based on MicroRNA Expression in Oral Squamous Cell Carcinoma

As described above, a variety of tumor-specific dysregulated miRNAs have been identified in OSCC, with either tumor suppressor or oncogenic functions. However, the challenges that remain for therapeutic application of miRNAs in OSCC are as follows: (a) miRNA selection, (b) complex regulatory mechanisms, (c) delivery, (d) pharmacokinetics, and (e) toxicity. Nevertheless, being endogenous molecules, miRNAs exhibit low toxicity in humans. Further, owing to their small size, miRNAs can be introduced into the system through different delivery methods.

In this context, so far, miRNA sponging, locked nucleic acid-mediated suppression of oncogenic miRNAs, and replacement of tumor-suppressive miRNAs using respective mimics/viral vectors/small compounds have already been used for different cancers (17, 119). To this end, the efficacy and accuracy of the miRNA delivery system are very important. Two main miRNA delivery approaches have been described: local (intra-tumor) and systemic. Systemic approaches would be suitable for metastatic or late-stage advanced cancers. The miRNA could be conjugated with a folate ligand, such as vitamin B9, for selective delivery to treat the cancer (17, 119). In addition, exosome/microvesicle/liposome-mediated delivery of miRNAs could also be used as novel tools for miRNA-based cancer therapy (53). Interestingly, therapeutic delivery of miRNA may be possible just through oral intake of vegetables, since it has been found that humans and animals can acquire plant miRNAs in their sera or body fluids through food intake (120). The first miRNA-based therapy specifically for cancer is MRX34, wherein a synthetic miR-34a mimic is loaded into liposomal nanoparticles (121). Quantification of MRX34 in non-human primates has established a satisfactory 7.7 h half-life in whole blood (122). Currently, only two observational miRNA-based therapies, miR-29b and miR-29, have progressed to clinical trials (Trial ref No.: NCT02009852 and NCT01927354, respectively) for OSCC. Further studies on miRNA-based diagnostics and therapies need to be evaluated extensively for OSCC treatment.

## Conclusion

This review highlighted the functional landscape of dysregulated miRNAs in OSCC from a clinical perspective. We identified 17 miRNAs (let-7d, miR-1, miR-125b-5p, miR-138-5p, miR-142, miR-145, miR-155, miR-16, miR-196a, miR-196b, miR-200c, miR-20a-5p, miR-21-5p, miR-218, miR-31-5p, miR-34a, and miR-375) commonly dysregulated in OSCC and that have been found to have clinical significance in three or more extensive studies. We also found 22 miRNAs (let-7d, miR-125b-5p, miR-145, miR-146a, miR-150, miR-16, miR-184, miR-191, miR-196a, miR-196b, miR-21-5p, miR-223, miR-24, miR-26a, miR-27b, miR-29a, miR-31-5p, miR-32-5p, miR-375, miR-451, miR-9 and miR-99b-3p) to be significantly dysregulated in two or more clinical sample types (tumor tissues/epithelial cells and one or more circulating body fluid) collected from OSCC patients. These miRNAs could have the potential for clinical application for disease diagnosis, patient stratification, and therapy in OSCC. Six miRNAs (miR-146a, miR-148a, miR-24, miR-438-5p, miR-9, and miR-99b-3p) which are common to different types of biological fluid samples (blood/plasma/serum/saliva) from OSCC patients could be potential biomarkers through minimally invasive or non-invasive methods to predict OSCC more accurately. Logical selection, validation, and confirmation of these potential miRNA biomarkers (single/panel) are very important for augmenting their specific clinical applications in OSCC.

The number of human miRNAs (>2,600) in miRbase significantly increases in every successive version of the database (recent is v22.1) due to continuous inclusion of novel miRNAs (123). In most of the cases, the newer studies came up with new sets of dysregulated miRNA signatures for OSCC detection and prognostication. Therefore, large differences are frequently found in the results of similar older studies and current large-scale data sets. The ethnicity of the recruited patient population is also an important issue in this situation. In the current review, importantly, we summarized the recent progress on elucidating the clinical significance of miRNAs (tumor-associated or circulating), especially with respect to possible ways to develop miRNA-based detection and prognostication methods in conjunction with available techniques. Recent evidence increasingly demonstrates that cell-free miRNAs are evolving as consistent and reliable biomarkers for early detection, disease monitoring, and patient stratification, as well as guides to optimum treatment protocols for patients with OSCC.

In conclusion, a wide variety of dysregulated miRNAs contribute to the OSCC phenotype and differential patient outcomes, including tumor progression, therapy response, recurrence, metastasis, and survival. Moreover, miRNA-mediated regulatory mechanisms are complex and tangled with numerous interconnected physiological events. Here, one of the biggest challenges is to identify the tailor-made potentially relevant key miRNA candidates (single or in spectrum) along with or without their key targets for detection of disease and stratification of each patient with OSCC. Therefore, on the basis of our previous knowledge, careful, logical selection, and functional characterization of signature miRNAs (mentioned above) are very important. Standardized validation studies must be undertaken to ensure the sensitivity, specificity, and robustness of the signature miRNAs for individual patient conditions. Thus, well-designed, multicentered, prospective trials with large patient cohorts would be necessary to mitigate external variations in data sets. This will provide useful information for molecular diagnostics and determination of prognostic information for improved management of OSCC.

## Author Contributions

RG conceived the idea, collected all data, and designed and wrote the manuscript. SR and AP supervised and contributed with their expert comments and views in the logical presentation of manuscript and checked and edited the manuscript. All authors approved the final manuscript.

## Conflict of Interest

The authors declare that the research was conducted in the absence of any commercial or financial relationships that could be construed as a potential conflict of interest.

## References

[1] Bray F Ferlay J Soerjomataram I Siegel RL Torre LA Jemal A. Global cancer statistics 2018: GLOBOCAN estimates of incidence and mortality worldwide for 36 cancers in 185 countries. CA Cancer J Clin. (2018) 68:394–424. 10.3322/caac.21492 30207593

[2] Ferlay J Colombet M Soerjomataram I Mathers C Parkin DM Pineros M. Estimating the global cancer incidence and mortality in 2018: GLOBOCAN sources and methods. Int J Cancer. (2019) 144:1941–53. 10.1002/ijc.31937 30350310

[3] International Agency for Research on Cancer W India, Source: Globacon 2018. The Global Cancer Observatory (2019). Available online at: https://gco.iarc.fr/today/data/factsheets/populations/356-india-fact-sheets.pdf

[4] Leemans CR Braakhuis BJ Brakenhoff RH. The molecular biology of head and neck cancer. Nat Rev Cancer. (2011) 11:9–22. 10.1038/nrc2982 21160525

[5] Rischin D Ferris RL Le QT. Overview of advances in head and neck cancer. J Clin Oncol. (2015) 33:3225–6. 10.1200/JCO.2015.63.6761 26351331

[6] Chinn SB Myers JN. Oral cavity carcinoma: current management, controversies, and future directions. J Clin Oncol. (2015) 33:3269–76. 10.1200/JCO.2015.61.2929 26351335 PMC5320919

[7] Koyfman SA Ismaila N Crook D D'Cruz AL. Management of the neck in squamous cell carcinoma of the oral cavity and oropharynx: ASCO Clinical Practice Guideline. J Clin Oncol. (2019) 37:1753–74. 10.1200/JCO.18.01921 30811281 PMC7098829

[8] Wang Z Jiang L Huang C Li Z Chen L Gou L. Comparative proteomics approach to screening of potential diagnostic and therapeutic targets for oral squamous cell carcinoma. Mol Cell Proteomics. (2008) 7:1639–50. 10.1074/mcp.M700520-MCP200 18458027 PMC2556024

[9] Schaaij-Visser TB Brakenhoff RH Leemans CR Heck AJ Slijper M. Protein biomarker discovery for head and neck cancer. J Proteomics. (2010) 73:1790–803. 10.1016/j.jprot.2010.01.013 20139037

[10] Kang H Kiess A Chung CH. Emerging biomarkers in head and neck cancer in the era of genomics. Nat Rev Clin Oncol. (2014) 12:11–26. 10.1038/nrclinonc.2014.192 25403939

[11] Wang Y Springer S Mulvey CL Silliman N Schaefer J Sausen M. Detection of somatic mutations and HPV in the saliva and plasma of patients with head and neck squamous cell carcinomas. Sci Transl Med. (2015) 7:293ra104. 10.1126/scitranslmed.aaa8507 26109104 PMC4587492

[12] Cohen JD Javed AA Thoburn C Wong F Tie J Gibbs P. Combined circulating tumor DNA and protein biomarker-based liquid biopsy for the earlier detection of pancreatic cancers. Proc Natl Acad Sci USA. (2017) 114:10202–7. 10.1073/pnas.1704961114 28874546 PMC5617273

[13] Cohen JD Li L Wang Y Thoburn C Afsari B Danilova L. Detection and localization of surgically resectable cancers with a multi-analyte blood test. Science. (2018) 359:926–30. 10.1126/science.aar3247 29348365 PMC6080308

[14] Wang K Zhang S Weber J Baxter D Galas DJ. Export of microRNAs and microRNA-protective protein by mammalian cells. Nucleic Acids Res. (2010) 38:7248–59. 10.1093/nar/gkq601 20615901 PMC2978372

[15] Arroyo JD Chevillet JR Kroh EM Ruf IK Pritchard CC Gibson DF. Argonaute2 complexes carry a population of circulating microRNAs independent of vesicles in human plasma. Proc Natl Acad Sci USA. (2011) 108:5003–8. 10.1073/pnas.1019055108 21383194 PMC3064324

[16] Vickers KC Palmisano BT Shoucri BM Shamburek RD Remaley AT. MicroRNAs are transported in plasma and delivered to recipient cells by high-density lipoproteins. Nat Cell Biol. (2011) 13:423–33. 10.1038/ncb2210 21423178 PMC3074610

[17] Iorio MV Croce CM. MicroRNA dysregulation in cancer: diagnostics, monitoring and therapeutics. A comprehensive review. EMBO Mol Med. (2012) 4:143–59. 10.1002/emmm.201100209 22351564 PMC3376845

[18] Dhawan A Scott JG Harris AL Buffa FM. Pan-cancer characterisation of microRNA across cancer hallmarks reveals microRNA-mediated downregulation of tumour suppressors. Nat Commun. (2018) 9:5228. 10.1038/s41467-018-07657-1 30531873 PMC6286392

[19] Lee RC Feinbaum RL Ambros V. The C. elegans heterochronic gene lin-4 encodes small RNAs with antisense complementarity to lin-14. Cell. (1993) 75:843–54. 10.1016/0092-8674(93)90529-Y 8252621

[20] Ebert MS Sharp PA. Roles for microRNAs in conferring robustness to biological processes. Cell. (2012) 149:515–24. 10.1016/j.cell.2012.04.005 22541426 PMC3351105

[21] Krol J Loedige I Filipowicz W. The widespread regulation of microRNA biogenesis, function and decay. Nat Rev Genet. (2010) 11:597–610. 10.1038/nrg2843 20661255

[22] Djuranovic S Nahvi A Green R. miRNA-mediated gene silencing by translational repression followed by mRNA deadenylation and decay. Science. (2012) 336:237–40. 10.1126/science.1215691 22499947 PMC3971879

[23] Jonas S Izaurralde E. Towards a molecular understanding of microRNA-mediated gene silencing. Nat Rev Genet. (2015) 16:421–33. 10.1038/nrg3965 26077373

[24] Redis RS Calin S Yang Y You MJ Calin GA. Cell-to-cell miRNA transfer: from body homeostasis to therapy. Pharmacol Ther. (2012) 136:169–74. 10.1016/j.pharmthera.2012.08.003 22903157 PMC3855335

[25] Fang C Li Y. Prospective applications of microRNAs in oral cancer. Oncol Lett. (2019) 18:3974–84. 10.3892/ol.2019.10751 31579085 PMC6757290

[26] Calin GA Dumitru CD Shimizu M Bichi R Zupo S Noch E. Frequent deletions and down-regulation of micro- RNA genes miR15 and miR16 at 13q14 in chronic lymphocytic leukemia. Proc Natl Acad Sci USA. (2002) 99:15524–9. 10.1073/pnas.242606799 12434020 PMC137750

[27] Hanahan D Weinberg RA. Hallmarks of cancer: the next generation. Cell. (2011) 144:646–74. 10.1016/j.cell.2011.02.013 21376230

[28] Bracken CP Scott HS Goodall GJ. A network-biology perspective of microRNA function and dysfunction in cancer. Nat Rev Genet. (2016) 17:719–32. 10.1038/nrg.2016.134 27795564

[29] Anastasiadou E Jacob LS Slack FJ. Non-coding RNA networks in cancer. Nat Rev Cancer. (2018) 18:5–18. 10.1038/nrc.2017.99 29170536 PMC6337726

[30] Cai Y Wan J. Competing endogenous RNA regulations in neurodegenerative disorders: current challenges and emerging insights. Front Mol Neurosci. (2018) 11:370. 10.3389/fnmol.2018.00370 30344479 PMC6182084

[31] Xie B Ding Q Han H Wu D. miRCancer: a microRNA-cancer association database constructed by text mining on literature. Bioinformatics. (2013) 29:638–44. 10.1093/bioinformatics/btt014 23325619

[32] Tran N Mclean T Zhang X Zhao CJ Thomson JM O'brien C. MicroRNA expression profiles in head and neck cancer cell lines. Biochem Biophys Res Commun. (2007) 358:12–7. 10.1016/j.bbrc.2007.03.201 17475218

[33] Chang SS Jiang WW Smith I Poeta LM Begum S Glazer C. MicroRNA alterations in head and neck squamous cell carcinoma. Int J Cancer. (2008) 123:2791–7. 10.1002/ijc.23831 18798260 PMC3184846

[34] Wong TS Liu XB Wong BY Ng RW Yuen AP Wei WI. Mature miR-184 as potential oncogenic microRNA of squamous cell carcinoma of tongue. Clin Cancer Res. (2008) 14:2588–92. 10.1158/1078-0432.CCR-07-0666 18451220

[35] Childs G Fazzari M Kung G Kawachi N Brandwein-Gensler M Mclemore M Low-level expression of microRNAs let-7d and miR-205 are prognostic markers of head and neck squamous cell carcinoma. Am J Pathol. (2009) 174:736–45. 10.2353/ajpath.2009.080731 19179615 PMC2665736

[36] Gombos K Horvath R Szele E Juhasz K Gocze K Somlai K. miRNA expression profiles of oral squamous cell carcinomas. Anticancer Res. (2013) 33:1511–7. Available online at: http://ar.iiarjournals.org/content/33/4/1511.full.pdf 23564792

[37] Hui AB Lenarduzzi M Krushel T Waldron L Pintilie M Shi W. Comprehensive MicroRNA profiling for head and neck squamous cell carcinomas. Clin Cancer Res. (2010) 16:1129–39. 10.1158/1078-0432.CCR-09-2166 20145181

[38] Lu YC Chen YJ Wang HM Tsai CY Chen WH Huang YC. Oncogenic function and early detection potential of miRNA-10b in oral cancer as identified by microRNA profiling. Cancer Prev Res. (2012) 5:665–74. 10.1158/1940-6207.CAPR-11-0358 22318752

[39] Kalfert D Pesta M Kulda V Topolcan O Ryska A Celakovsky P. MicroRNA profile in site-specific head and neck squamous cell cancer. Anticancer Res. (2015) 35:2455–63. Available online at: http://ar.iiarjournals.org/content/35/4/2455.full.pdf 25862914

[40] Manikandan M Deva Magendhra Rao AK Arunkumar G Manickavasagam M Rajkumar KS Rajaraman R. Oral squamous cell carcinoma: microRNA expression profiling and integrative analyses for elucidation of tumourigenesis mechanism. Mol Cancer. (2016) 15:28. 10.1186/s12943-016-0512-8 27056547 PMC4823852

[41] Lamperska KM Kozlowski P Kolenda T Teresiak A Blizniak R Przybyla W. Unpredictable changes of selected miRNA in expression profile of HNSCC. Cancer Biomark. (2016) 16:55–64. 10.3233/CBM-150540 26484611 PMC13016531

[42] Zeljic K Jovanovic I Jovanovic J Magic Z Stankovic A Supic G. MicroRNA meta-signature of oral cancer: evidence from a meta-analysis. Ups J Med Sci. (2018) 123:43–9. 10.1080/03009734.2018.1439551 29482431 PMC5901467

[43] Towle R Gorenchtein M Garnis C Dickman C Zhu Y Poh CF Dysregulation of microRNAs across oral squamous cell carcinoma fields in non-smokers. J Interdiscipl Med Dent Sci. (2014) 2:131 10.4172/2376-032X.1000131

[44] Shiah SG Hsiao JR Chang WM Chen YW Jin YT Wong TY. Downregulated miR329 and miR410 promote the proliferation and invasion of oral squamous cell carcinoma by targeting Wnt-7b. Cancer Res. (2014) 74:7560–72. 10.1158/0008-5472.CAN-14-0978 25351956

[45] Hou YY Lee JH Chen HC Yang CM Huang SJ Liou HH. The association between miR-499a polymorphism and oral squamous cell carcinoma progression. Oral Dis. (2015) 21:195–206. 10.1111/odi.12241 24690080

[46] Lajer CB Garnaes E Friis-Hansen L Norrild B Therkildsen MH Glud M. The role of miRNAs in human papilloma virus (HPV)-associated cancers: bridging between HPV-related head and neck cancer and cervical cancer. Br J Cancer. (2012) 106:1526–34. 10.1038/bjc.2012.109 22472886 PMC3341860

[47] Network TCGA Comprehensive genomic characterization of head and neck squamous cell carcinomas. Nature. (2015) 517:576–82. 10.1038/nature14129 25631445 PMC4311405

[48] Hu J Ge W Xu J. HPV 16 E7 inhibits OSCC cell proliferation, invasion, and metastasis by upregulating the expression of miR-20a. Tumour Biol. (2016) 37:9433–40. 10.1007/s13277-016-4817-4 26781875

[49] Bozinovic K Sabol I Dediol E Milutin Gasperov N Manojlovic S Vojtechova Z. Genome-wide miRNA profiling reinforces the importance of miR-9 in human papillomavirus associated oral and oropharyngeal head and neck cancer. Sci Rep. (2019) 9:2306. 10.1038/s41598-019-38797-z 30783190 PMC6381209

[50] Gissi DB Morandi L Gabusi A Tarsitano A Marchetti C Cura F. A noninvasive test for microRNA expression in oral squamous cell carcinoma. Int J Mol Sci. (2018) 19:1789. 10.3390/ijms19061789 29914173 PMC6032413

[51] Adami GR Tang JL Markiewicz MR. Improving accuracy of RNA-based diagnosis and prognosis of oral cancer by using noninvasive methods. Oral Oncol. (2017) 69:62–7. 10.1016/j.oraloncology.2017.04.001 28559022 PMC5497764

[52] Zhou Y Kolokythas A Schwartz JL Epstein JB Adami GR. microRNA from brush biopsy to characterize oral squamous cell carcinoma epithelium. Cancer Med. (2017) 6:67–78. 10.1002/cam4.951 27989009 PMC5275769

[53] Kamerkar S Lebleu VS Sugimoto H Yang S Ruivo CF Melo SA. Exosomes facilitate therapeutic targeting of oncogenic KRAS in pancreatic cancer. Nature. (2017) 546:498–503. 10.1038/nature22341 28607485 PMC5538883

[54] Redova M Sana J Slaby O. Circulating miRNAs as new blood-based biomarkers for solid cancers. Future Oncol. (2013) 9:387–402. 10.2217/fon.12.192 23469974

[55] Mitchell PS Parkin RK Kroh EM Fritz BR Wyman SK Pogosova-Agadjanyan EL. Circulating microRNAs as stable blood-based markers for cancer detection. Proc Natl Acad Sci USA. (2008) 105:10513–8. 10.1073/pnas.0804549105 18663219 PMC2492472

[56] Turchinovich A Weiz L Langheinz A Burwinkel B. Characterization of extracellular circulating microRNA. Nucleic Acids Res. (2011) 39:7223–33. 10.1093/nar/gkr254 21609964 PMC3167594

[57] Rapado-Gonzalez O Lopez-Lopez R Lopez-Cedrun JL Triana-Martinez G Muinelo-Romay L Suarez-Cunqueiro MM. Cell-free microRNAs as potential oral cancer biomarkers: from diagnosis to therapy. Cells. (2019) 8:1653. 10.3390/cells8121653 31861130 PMC6952938

[58] Park NJ Zhou H Elashoff D Henson BS Kastratovic DA Abemayor E. Salivary microRNA: discovery, characterization, and clinical utility for oral cancer detection. Clin Cancer Res. (2009) 15:5473–7. 10.1158/1078-0432.CCR-09-0736 19706812 PMC2752355

[59] Yang Y Li YX Yang X Jiang L Zhou ZJ Zhu YQ. Progress risk assessment of oral premalignant lesions with saliva miRNA analysis. BMC Cancer. (2013) 13:129. 10.1186/1471-2407-13-129 23510112 PMC3637283

[60] Momen-Heravi F Trachtenberg AJ Kuo WP Cheng YS. Genomewide study of salivary microRNAs for detection of oral cancer. J Dent Res. (2014) 93:86S−93S. 10.1177/0022034514531018 24718111 PMC4107544

[61] Hung PS Liu CJ Chou CS Kao SY Yang CC Chang KW. miR-146a enhances the oncogenicity of oral carcinoma by concomitant targeting of the IRAK1, TRAF6 and NUMB genes. PLoS ONE. (2013) 8:e79926. 10.1371/journal.pone.0079926 24302991 PMC3841223

[62] Liu CJ Lin JS Cheng HW Hsu YH Cheng CY Lin SC. Plasma miR-187^*^ is a potential biomarker for oral carcinoma. Clin Oral Investig. (2016) 21:1131–8. 10.1007/s00784-016-1887-z 27324473

[63] Liu CJ Tsai MM Tu HF Lui MT Cheng HW Lin SC. miR-196a overexpression and miR-196a2 gene polymorphism are prognostic predictors of oral carcinomas. Ann Surg Oncol. (2013) 20(Suppl 3):S406–14. 10.1245/s10434-012-2618-6 23138850

[64] Lu YC Chang JT Huang YC Huang CC Chen WH Lee LY. Combined determination of circulating miR-196a and miR-196b levels produces high sensitivity and specificity for early detection of oral cancer. Clin Biochem. (2015) 48:115–21. 10.1016/j.clinbiochem.2014.11.020 25485932

[65] Sun G Cao Y Wang P Song H Bie T Li M. miR-200b-3p in plasma is a potential diagnostic biomarker in oral squamous cell carcinoma. Biomarkers. (2018) 23:137–41. 10.1080/1354750X.2017.1289241 28135849

[66] Chang YA Weng SL Yang SF Chou CH Huang WC Tu SJ. A three-microRNA signature as a potential biomarker for the early detection of oral cancer. Int J Mol Sci. (2018) 19:758. 10.3390/ijms19030758 29518940 PMC5877619

[67] Xu H Yang Y Zhao H Yang X Luo Y Ren Y. Serum miR-483-5p: a novel diagnostic and prognostic biomarker for patients with oral squamous cell carcinoma. Tumour Biol. (2016) 37:447–53. 10.1007/s13277-015-3514-z 26224475

[68] Sun L Liu L Fu H Wang Q Shi Y. Association of decreased expression of serum miR-9 with poor prognosis of oral squamous cell carcinoma patients. Med Sci Monit. (2016) 22:289–94. 10.12659/MSM.895683 26813876 PMC4734672

[69] Schneider A Victoria B Lopez YN Suchorska W Barczak W Sobecka A. Tissue and serum microRNA profile of oral squamous cell carcinoma patients. Sci Rep. (2018) 8:675. 10.1038/s41598-017-18945-z 29330429 PMC5766573

[70] Ren W Qiang C Gao L Li SM Zhang LM Wang XL. Circulating microRNA-21 (MIR-21) and phosphatase and tensin homolog (PTEN) are promising novel biomarkers for detection of oral squamous cell carcinoma. Biomarkers. (2014) 19:590–6. 10.3109/1354750X.2014.955059 25174622

[71] Ries J Vairaktaris E Agaimy A Kintopp R Baran C Neukam FW. miR-186, miR-3651 and miR-494: potential biomarkers for oral squamous cell carcinoma extracted from whole blood. Oncol Rep. (2014) 31:1429–36. 10.3892/or.2014.2983 24452363

[72] Ries J Baran C Wehrhan F Weber M Neukam FW Krautheim-Zenk A. Prognostic significance of altered miRNA expression in whole blood of OSCC patients. Oncol Rep. (2017) 37:3467–74. 10.3892/or.2017.5639 28498442

[73] Ries J Baran C Wehrhan F Weber M Motel C Kesting M. The altered expression levels of miR-186, miR-494 and miR-3651 in OSCC tissue vary from those of the whole blood of OSCC patients. Cancer Biomark. (2019) 24:19–30. 10.3233/CBM-180032 30594916 PMC13082507

[74] Mazumder S Datta S Ray JG Chaudhuri K Chatterjee R. Liquid biopsy: miRNA as a potential biomarker in oral cancer. Cancer Epidemiol. (2019) 58:137–45. 10.1016/j.canep.2018.12.008 30579238

[75] Blondal T Jensby Nielsen S Baker A Andreasen D Mouritzen P Wrang Teilum M Assessing sample and miRNA profile quality in serum and plasma or other biofluids. Methods. (2013) 59:S1–6. 10.1016/j.ymeth.2012.09.015 23036329

[76] Buschmann D Haberberger A Kirchner B Spornraft M Riedmaier I Schelling G. Toward reliable biomarker signatures in the age of liquid biopsies - how to standardize the small RNA-Seq workflow. Nucleic Acids Res. (2016) 44:5995–6018. 10.1093/nar/gkw545 27317696 PMC5291277

[77] Garcia-Elias A Alloza L Puigdecanet E Nonell L Tajes M Curado J. Defining quantification methods and optimizing protocols for microarray hybridization of circulating microRNAs. Sci Rep. (2017) 7:7725. 10.1038/s41598-017-08134-3 28798363 PMC5552704

[78] Gevaert AB Witvrouwen I Vrints CJ Heidbuchel H Van Craenenbroeck EM Van Laere SJ. MicroRNA profiling in plasma samples using qPCR arrays: recommendations for correct analysis and interpretation. PLoS ONE. (2018) 13:e0193173. 10.1371/journal.pone.0193173 29474497 PMC5825041

[79] Orsos Z Szanyi I Csejtei A Gerlinger I Ember I Kiss I. Association of pre-miR-146a rs2910164 polymorphism with the risk of head and neck cancer. Anticancer Res. (2013) 33:341–6. Available online at: http://ar.iiarjournals.org/content/33/1/341.full.pdf 23267167

[80] Tu HF Liu CJ Chang CL Wang PW Kao SY Yang CC. The association between genetic polymorphism and the processing efficiency of miR-149 affects the prognosis of patients with head and neck squamous cell carcinoma. PLoS ONE. (2012) 7:e51606. 10.1371/journal.pone.0051606 23272122 PMC3522737

[81] Niu YM Du XY Lu MY Xu QL Luo J Shen M. Significant association between functional microRNA polymorphisms and head and neck cancer susceptibility: a comprehensive meta-analysis. Sci Rep. (2015) 5:12972. 10.1038/srep17149 26277865 PMC4538372

[82] Brito JA Gomes CC Guimaraes AL Campos K Gomez RS. Relationship between microRNA expression levels and histopathological features of dysplasia in oral leukoplakia. J Oral Pathol Med. (2014) 43:211–6. 10.1111/jop.12112 24020903

[83] Kinoshita T Nohata N Hanazawa T Kikkawa N Yamamoto N Yoshino H. Tumour-suppressive microRNA-29s inhibit cancer cell migration and invasion by targeting laminin-integrin signalling in head and neck squamous cell carcinoma. Br J Cancer. (2013) 109:2636–45. 10.1038/bjc.2013.607 24091622 PMC3833206

[84] Tachibana H Sho R Takeda Y Zhang X Yoshida Y Narimatsu H. Circulating miR-223 in oral cancer: its potential as a novel diagnostic biomarker and therapeutic target. PLoS ONE. (2016) 11:e0159693. 10.1371/journal.pone.0159693 27441818 PMC4956265

[85] Ghosh RD Ghuwalewala S Das P Mandloi S Alam SK Chakraborty J. MicroRNA profiling of cisplatin-resistant oral squamous cell carcinoma cell lines enriched with cancer-stem-cell-like and epithelial-mesenchymal transition-type features. Sci Rep. (2016) 6:23932. 10.1038/srep23932 27045798 PMC4820705

[86] Zeng G Xun W Wei K Yang Y Shen H. MicroRNA-27a-3p regulates epithelial to mesenchymal transition via targeting YAP1 in oral squamous cell carcinoma cells. Oncol Rep. (2016) 36:1475–82. 10.3892/or.2016.4916 27432214

[87] Lu YC Chang JT Liao CT Kang CJ Huang SF Chen IH. OncomiR-196 promotes an invasive phenotype in oral cancer through the NME4-JNK-TIMP1-MMP signaling pathway. Mol Cancer. (2014) 13:218. 10.1186/1476-4598-13-218 25233933 PMC4176851

[88] Tu HF Chang KW Cheng HW Liu CJ. Upregulation of miR-372 and−373 associates with lymph node metastasis and poor prognosis of oral carcinomas. Laryngoscope. (2015) 125:E365–70. 10.1002/lary.25464 26152520

[89] Hou YY You JJ Yang CM Pan HW Chen HC Lee JH. Aberrant DNA hypomethylation of miR-196b contributes to migration and invasion of oral cancer. Oncol Lett. (2016) 11:4013–21. 10.3892/ol.2016.4491 27313732 PMC4888123

[90] Lin SC Kao SY Chang JC Liu YC Yu EH Tseng SH. Up-regulation of miR-187 modulates the advances of oral carcinoma by targeting BARX2 tumor suppressor. Oncotarget. (2016) 7:61355–65. 10.18632/oncotarget.11349 27542258 PMC5308656

[91] Chang CJ Hsu CC Chang CH Tsai LL Chang YC Lu SW. Let-7d functions as novel regulator of epithelial-mesenchymal transition and chemoresistant property in oral cancer. Oncol Rep. (2011) 26:1003–10. 10.3892/or.2011.1360 21725603

[92] Lei W Liu YE Zheng Y Qu L. MiR-429 inhibits oral squamous cell carcinoma growth by targeting ZEB1. Med Sci Monit. (2015) 21:383–9. 10.12659/MSM.893412 25640197 PMC4324578

[93] Wu B Lei D Wang L Yang X Jia S Yang Z. MiRNA-101 inhibits oral squamous-cell carcinoma growth and metastasis by targeting zinc finger E-box binding homeobox 1. Am J Cancer Res. (2016) 6:1396–407. Available online at: http://www.ajcr.us/files/ajcr0026756.pdf 27429852 PMC4937741

[94] Yu J Xie F Bao X Chen W Xu Q. miR-300 inhibits epithelial to mesenchymal transition and metastasis by targeting Twist in human epithelial cancer. Mol Cancer. (2014) 13:121. 10.1186/1476-4598-13-121 24885626 PMC4040483

[95] Bufalino A Cervigne NK De Oliveira CE Fonseca FP Rodrigues PC Macedo CC. Low miR-143/miR-145 cluster levels induce activin a overexpression in oral squamous cell carcinomas, which contributes to poor prognosis. PLoS ONE. (2015) 10:e0136599. 10.1371/journal.pone.0136599 26317418 PMC4552554

[96] Chang WM Lin YF Su CY Peng HY Chang YC Lai TC. Dysregulation of RUNX2/activin-A axis upon miR-376c downregulation promotes lymph node metastasis in head and neck squamous cell carcinoma. Cancer Res. (2016) 76:7140–50. 10.1158/0008-5472.CAN-16-1188 27760788

[97] Lu L Xue X Lan J Gao Y Xiong Z Zhang H. MicroRNA-29a upregulates MMP2 in oral squamous cell carcinoma to promote cancer invasion and anti-apoptosis. Biomed Pharmacother. (2014) 68:13–9. 10.1016/j.biopha.2013.10.005 24210072

[98] Fukumoto I Koshizuka K Hanazawa T Kikkawa N Matsushita R Kurozumi A. The tumor-suppressive microRNA-23b/27b cluster regulates the MET oncogene in oral squamous cell carcinoma. Int J Oncol. (2016) 49:1119–29. 10.3892/ijo.2016.3602 27573718

[99] Uesugi A Kozaki K Tsuruta T Furuta M Morita K Imoto I. The tumor suppressive microRNA miR-218 targets the mTOR component Rictor and inhibits AKT phosphorylation in oral cancer. Cancer Res. (2011) 71:5765–78. 10.1158/0008-5472.CAN-11-0368 21795477

[100] Kinoshita T Hanazawa T Nohata N Kikkawa N Enokida H Yoshino H. Tumor suppressive microRNA-218 inhibits cancer cell migration and invasion through targeting laminin-332 in head and neck squamous cell carcinoma. Oncotarget. (2012) 3:1386–400. 10.18632/oncotarget.709 23159910 PMC3717800

[101] Lin F Yao L Xiao J Liu D Ni Z. MiR-206 functions as a tumor suppressor and directly targets K-Ras in human oral squamous cell carcinoma. Onco Targets Ther. (2014) 7:1583–91. 10.2147/OTT.S67624 25246801 PMC4166217

[102] Koshizuka K Hanazawa T Fukumoto I Kikkawa N Matsushita R Mataki H. Dual-receptor (EGFR and c-MET) inhibition by tumor-suppressive miR-1 and miR-206 in head and neck squamous cell carcinoma. J Hum Genet. (2017). 62:113–21. 10.1038/jhg.2016.47 27169691

[103] Bonnin N Armandy E Carras J Ferrandon S Battiston-Montagne P Aubry M. MiR-422a promotes loco-regional recurrence by targeting NT5E/CD73 in head and neck squamous cell carcinoma. Oncotarget. (2016) 7:44023–38. 10.18632/oncotarget.9829 27281619 PMC5190076

[104] Liu CJ Shen WG Peng SY Cheng HW Kao SY Lin SC. miR-134 induces oncogenicity and metastasis in head and neck carcinoma through targeting WWOX gene. Int J Cancer. (2014) 134:811–21. 10.1002/ijc.28358 23824713

[105] Henson BJ Bhattacharjee S O'dee DM Feingold E Gollin SM. Decreased expression of miR-125b and miR-100 in oral cancer cells contributes to malignancy. Genes Chromosomes Cancer. (2009) 48:569–82. 10.1002/gcc.20666 19396866 PMC2726991

[106] Shiiba M Shinozuka K Saito K Fushimi K Kasamatsu A Ogawara K. MicroRNA-125b regulates proliferation and radioresistance of oral squamous cell carcinoma. Br J Cancer. (2013) 108:1817–21. 10.1038/bjc.2013.175 23591197 PMC3658524

[107] Zhou X Ren Y Liu A Jin R Jiang Q Huang Y. WP1066 sensitizes oral squamous cell carcinoma cells to cisplatin by targeting STAT3/miR-21 axis. Sci Rep. (2014) 4:7461. 10.1038/srep07461 25514838 PMC4268632

[108] Suh YE Raulf N Gaken J Lawler K Urbano TG Bullenkamp J. MicroRNA-196a promotes an oncogenic effect in head and neck cancer cells by suppressing annexin A1 and enhancing radioresistance. Int J Cancer. (2015) 137:1021–34. 10.1002/ijc.29397 25523631

[109] Kawahara K Nakayama H Nagata M Yoshida R Hirosue A Tanaka T. A low Dicer expression is associated with resistance to 5-FU-based chemoradiotherapy and a shorter overall survival in patients with oral squamous cell carcinoma. J Oral Pathol Med. (2014) 43:350–6. 10.1111/jop.12140 24325353

[110] Yu ZW Zhong LP Ji T Zhang P Chen WT Zhang CP. MicroRNAs contribute to the chemoresistance of cisplatin in tongue squamous cell carcinoma lines. Oral Oncol. (2010) 46:317–22. 10.1016/j.oraloncology.2010.02.002 20219416

[111] Lin J Lin Y Fan L Kuang W Zheng L Wu J. miR-203 inhibits cell proliferation and promotes cisplatin induced cell death in tongue squamous cancer. Biochem Biophys Res Commun. (2016) 473:382–7. 10.1016/j.bbrc.2016.02.105 26946357

[112] Sun L Yao Y Liu B Lin Z Lin L Yang M. MiR-200b and miR-15b regulate chemotherapy-induced epithelial-mesenchymal transition in human tongue cancer cells by targeting BMI1. Oncogene. (2012) 31:432–45. 10.1038/onc.2011.263 21725369

[113] Liao L Wang J Ouyang S Zhang P Wang J Zhang M. Expression and clinical significance of microRNA-1246 in human oral squamous cell carcinoma. Med Sci Monit. (2015) 21:776–81. 10.12659/MSM.892508 25791131 PMC4371709

[114] Guan GF Zhang DJ Wen LJ Xin D Liu Y Yu DJ. Overexpression of lncRNA H19/miR-675 promotes tumorigenesis in head and neck squamous cell carcinoma. Int J Med Sci. (2016) 13:914–22. 10.7150/ijms.16571 27994496 PMC5165684

[115] Xu P Li Y Zhang H Li M Zhu H. MicroRNA-340 mediates metabolic shift in oral squamous cell carcinoma by targeting glucose transporter-1. J Oral Maxillofac Surg. (2016) 74:844–50. 10.1016/j.joms.2015.09.038 26541225

[116] Qiao B Cai JH King-Yin Lam A He BX. MicroRNA-542-3p inhibits oral squamous cell carcinoma progression by inhibiting ILK/TGF-beta1/Smad2/3 signaling. Oncotarget. (2017) 8:70761–76. 10.18632/oncotarget.19986 29050317 PMC5642592

[117] Zhang B Li Y Hou D Shi Q Yang S Li Q. MicroRNA-375 inhibits growth and enhances radiosensitivity in oral squamous cell carcinoma by targeting insulin like growth factor 1 receptor. Cell Physiol Biochem. (2017) 42:2105–17. 10.1159/000479913 28810236

[118] Koshizuka K Hanazawa T Kikkawa N Katada K Okato A Arai T. Antitumor miR-150-5p and miR-150-3p inhibit cancer cell aggressiveness by targeting SPOCK1 in head and neck squamous cell carcinoma. Auris Nasus Larynx. (2018) 45:854–65. 10.1016/j.anl.2017.11.019 29233721

[119] Rupaimoole R Slack FJ. MicroRNA therapeutics: towards a new era for the management of cancer and other diseases. Nat Rev Drug Discov. (2017) 16:203–22. 10.1038/nrd.2016.246 28209991

[120] Zhang L Hou D Chen X Li D Zhu L Zhang Y. Exogenous plant MIR168a specifically targets mammalian LDLRAP1: evidence of cross-kingdom regulation by microRNA. Cell Res. (2012) 22:107–26. 10.1038/cr.2011.158 21931358 PMC3351925

[121] Bouchie A. First microRNA mimic enters clinic. Nat Biotechnol. (2013) 31:577. 10.1038/nbt0713-577 23839128

[122] Kelnar K Peltier HJ Leatherbury N Stoudemire J Bader AG. Quantification of therapeutic miRNA mimics in whole blood from nonhuman primates. Anal Chem. (2014) 86:1534–42. 10.1021/ac403044t 24397447 PMC3982984

[123] Griffiths-Jones S Saini HK Van Dongen S Enright AJ. miRBase: tools for microRNA genomics. Nucleic Acids Res. (2008) 36:D154–8. 10.1093/nar/gkm952 17991681 PMC2238936

