# Supplementary material for: Functional Landscape of Dysregulated MicroRNAs in Oral Squamous Cell Carcinoma: Clinical Implications
Source: Front Oncol. 2020 May 12;10:619. doi: 10.3389/fonc.2020.00619 (PMC7274490; doi:10.3389/fonc.2020.00619)
Supplement: Supplementary file 1 [file Data_Sheet_1.PDF]

Supplementary Material

Functional landscape of dysregulated microRNAs in oral squamous cell carcinoma: Clinical implications

Ruma Dey Ghosh[1]*, Pattathyeil Arun[2] and Susanta Roychoudhury[3]

[1]Tata Translational Cancer Research Centre, Tata Medical Centre

[2]Department of Head and Neck Surgical Oncology, Tata Medical Centre

[3]Saroj Gupta Cancer Centre and Research Institute

*Correspondence: Dr. Ruma Dey Ghosh, Tata Translational Cancer Research Centre, Tata Medical Centre, 14 MAR (E-W), New Town, Rajarhut, Kolkata -700156, India. E-mail: deyrumai@yahoo.co.in, ruma.deyghosh@tmckolkata.com

Supplementary Table 1: List of dysregulated microRNAs (miRNAs) in oral squamous cell carcinoma (OSCC).

miRNA Id	Profile status in OSCC	Sample type	Clinical sample source	Targets	Impact on	References
**hsa-let-7a**	Downregulated	Clinical	Tumor		PI3K/Akt & p53 signaling	(Manikandan et al., 2016)
**hsa-let-7a-3p**	Upregulated	Clinical	Tumor			(Schneider et al., 2018)
**hsa-let-7c-5p**	Downregulated	Clinical	Tumor			(Schneider et al., 2018)
**hsa-let-7d**	Downregulated	Clinical/ Primary culture/ Cell line	Tumor/Serum	Twist and Snail	EMT, invasion, chemo-resistance & poor survival	(Childs et al., 2009;Chang et al., 2011;Manikandan et al., 2016)
**hsa-let-7f**	Downregulated	Clinical	Tumor		PI3K/Akt & p53 signaling	(Manikandan et al., 2016)
**hsa-let-7i**	Upregulated	Clinical/Cell line	Tumor			(Hui et al., 2010)
**hsa-miR-1**	Downregulated	Clinical/Cell line	Tumor	TAGLN2, EGFR, c-MET, Slug	Aggressiveness & Invasion	(Childs et al., 2009;Nohata et al., 2011;Koshizuka et al., 2016)
**hsa-miR-100**	Downregulated	Clinical	Tumor		Loss of sensitivity to ionizing radiation	(Henson et al., 2009)
**hsa-miR-106b**	Upregulated	Clinical/Cell line	Tumor			(Hui et al., 2010)
**hsa-miR-107**	Downregulated	Clinical/Cell line	Tumor	PKCε, CDK6, HIF1-β	Invasion	(Datta et al., 2012;Piao et al., 2012)
**hsa-miR-10a**	Downregulated	Clinical/Cell line	Tumor			(Hui et al., 2010)
**hsa-miR-10b**	Upregulated	Cell line			Migration and invasion	(Lu et al., 2012)
**hsa-miR-124**	Downregulated	Cell line		ITGB1	Invasion	(Hunt et al., 2011)
**hsa-miR-1246**	Upregulated	Clinical/Cell line	Tumor		Proliferation, invasion, metastasis, clinical stage, LNM, poor survival	(Liao et al., 2015)
**hsa-miR-1250**	Downregulated	Clinical	Saliva			(Momen-Heravi et al., 2014)
**hsa-miR-125b-5p**	Downregulated	Clinical/Cell line	Tumor / Plasma		Loss of sensitivity to ionizing radiation	(Henson et al., 2009;Hui et al., 2010;Gu et al., 2015;Manikandan

						et al., 2015b;Schneider et al., 2018)
**hsa-miR-125b-2-3p**	Downregulated	Clinical	Tumor			(Schneider et al., 2018)
**hsa-miR-126**	Downregulated	Clinical/Cell line	Tumor	EGFL7, VEGF, bFGF	Invasion, nodal metastasis, poor prognosis, survival	(Sasahira et al., 2012;Yang et al., 2014)
**hsa-miR-127**	Upregulated	Clinical	Tumor		Tumor progression	(Wiklund et al., 2011)
**hsa-miR-1271**	Downregulated	Clinical/Cell line	Tumor	ALK	Metastasis	(Kong et al., 2015)
**hsa-miR-1275**	Upregulated	Clinical	Tumor		Lymph node invasion	(Manikandan et al., 2016)
**hsa-miR-128**	Downregulated	Cell line		BMI1, BAG2, BAX, H3f3b, Paip2	Tumorigenesis and progression	(Hauser et al., 2015)
**hsa-miR-128a**	Upregulated	Cell line				(Lu et al., 2012)
**hsa-miR-1291**	Downregulated	Clinical	Tumor			(Schneider et al., 2018)
**hsa-miR-133a**	Downregulated	Clinical/Cell line	Tumor	MSN	Invasion	(Childs et al., 2009;Kinoshita et al., 2012b)
**hsa-miR-133a-3p**	Downregulated	Clinical/Cell line	Tumor	COL1A1	Proliferation, migration, invasion	(He et al., 2018)
**hsa-miR-134**	Upregulated	Clinical/Cell line	Plasma	WWOX	Metastasis & poor survival	(Liu et al., 2014)
**hsa-miR-135b-5p**	Upregulated	Clinical	Tumor			(Schneider et al., 2018)
**hsa-miR-136**	Downregulated	Clinical	Saliva			(Momen-Heravi et al., 2014)
**hsa-miR-137**	Downregulated	Clinical/Cell line	Tumor		Invasion, metastasis	(Sun et al., 2018b)
**hsa-miR-138**	Downregulated	Clinical/Cell line	Tumor	CLNSIA, GNAI2, SLC20A1, RhoC, YAP1	Invasion & metastasis	(Liu et al., 2009;Jin et al., 2013;Islam et al., 2014;Manikandan et al., 2015b;Xu et al., 2015)
**hsa-miR-138-5p**	Downregulated	Clinical/Cell line	Tumor	ΔNP63, SOX2, CD44, NOTCH1, KLF4	Growth, metastasis, stemness	(Zhuang et al., 2017)
**hsa-miR-139-5p**	Downregulated	Clinical	Tumor			(Schneider et al., 2018)
**hsa-miR-141**	Downregulated	Clinical	Tumor			(Chen et al., 2016)
**hsa-miR-142-3p**	Upregulated	Clinical/Cell line	Tumor		PI3K/Akt & p53 signaling	(Hui et al., 2010;Manikandan et al., 2016;Schneider et al., 2018)
**hsa-miR-142-5p**	Upregulated	Clinical	Tumor			(Schneider et al., 2018)
**hsa-miR-143**	Downregulated	Clinical/Cell line	Tumor	Activin A, Hexokinase	Proliferation, EMT, Invasion & metastasis	(Bufalino et al., 2015;Manikandan et al., 2015a)
**hsa-miR-144**	Upregulated	Clinical	Tumor		PI3K/Akt & p53 signaling	(Manikandan et al., 2016)
**hsa-miR-145**	Downregulated	Clinical/Cell line	Tumor / Saliva	Activin A, c-MYC, CDK6	EMT, Invasion & metastasis, early tumorigenesis	(Gao et al., 2013;Shao et al., 2013;Bufalino et al., 2015)
**hsa-miR-146a**	Upregulated	Clinical	Plasma/ Saliva/ Brush biopsy	IRAK1, TRAF6, NUMB	Carcinogenesis	(Hung et al., 2013;Gissi et al., 2018)
**hsa-miR-146b**	Upregulated	Clinical	Tumor			(Chang et al., 2008)
**hsa-miR-147**	Downregulated	Clinical	Saliva			(Momen-Heravi et al., 2014)
**hsa-miR-148a**	Downregulated	Clinical	Plasma / Saliva			(Momen-Heravi et al., 2014)
**hsa-miR-148b**	Upregulated	Cell line				(Lu et al., 2012)
**hsa-miR-149**	Downregulated	Clinical	Tumor		tumor progression & poor patient survival	(Tu et al., 2012)
**hsa-miR-150-3p**	Downregulated	Clinical/Cell line	Tumor	SPOCK1	Aggressiveness	(Koshizuka et al., 2018)

**hsa-miR-150-5p**	Upregulated	Clinical	Tumor/ Plasma	SPOCK1	Aggressiveness	(Chang et al., 2018;Koshizuka et al., 2018)
**hsa-miR-155**	Upregulated	Clinical/Cell line	Tumor	p27kip1	Proliferation, poor prognosis	(Hui et al., 2010;Ni et al., 2014;Manikandan et al., 2015b;Shi et al., 2015;Manikandan et al., 2016)
**hsa-miR-15b**	Upregulated	Clinical/Cell line	Tumor	BMI1	EMT, chemoresistance, poor survival	(Lu et al., 2012)
**hsa-miR-16**	Up/Downregulated	Clinical/Cell line	Tumor / Serum		PI3K/Akt & p53 signaling	(Hui et al., 2010;Kimura et al., 2010;Manikandan et al., 2016)
**hsa-miR-181a**	Downregulated	Clinical/Cell line	Tumor	KRAS		(Shin et al., 2011)
**hsa-miR-181b**	Upregulated	Clinical	Saliva		Grade dysplasia	(Brito et al., 2014)
**hsa-miR-184**	Downregulated	Clinical/Cell line	Tumor / Plasma /Serum/ Saliva	FIH	Tumorigenesis	(Manikandan et al., 2015b;Kao et al., 2016)
**hsa-miR-186**	Downregulated	Clinical/Cell line	Tumor	PTEN, SHP2, ERK, AKT signaling	Aggressiveness, proliferation	(Cai et al., 2018)
**hsa-miR-187**	Upregulated	Cell line	Plasma	BARX2	Nodal metastasis	(Lin et al., 2016)
**hsa-miR-187-3p**	Upregulated	Clinical/Cell line	Tumor		Prognostic factor	(Liu et al., 2016;Schneider et al., 2018)
**hsa-miR-188**	Downregulated	Clinical	Tumor	SIX1	Progression & invasion	(Wang and Liu, 2016)
**hsa-miR-18a**	Upregulated	Clinical	Tumor			(Schneider et al., 2018)
**hsa-miR-191**	Upregulated	Clinical	Tumor/ Brush biopsy			(Gombos et al., 2013;Gissi et al., 2018)
**hsa-miR-195-5p**	Downregulated	Clinical/Cell line	Serum	TRIM14	Proliferation, migration, invasion	(Wang et al., 2017)
**hsa-miR-196a**	Upregulated	Clinical/Cell line	Tumor / Plasma	ANXA1	Poor prognosis & radio resistance; Nodal metastasis & NME4-JNK-TIMP-MMP signaling	(Lu et al., 2012;Liu et al., 2013;Lu et al., 2014b;Suh et al., 2015)
**hsa-miR-196b-5p**	Upregulated	Clinical/Cell line	Tumor / Plasma		Nodal metastasis & NME4-JNK-TIMP-MMP signaling	(Lu et al., 2012;Lu et al., 2014b;Hou et al., 2016;Schneider et al., 2018)
**hsa-miR-19a-3p**	Upregulated	Clinical	Tumor			(Schneider et al., 2018)
**hsa-miR-200a**	Downregulated	Clinical/Cell line	Tumor	GRHL2	Pro-tumorigenic	(Chen et al., 2016)
**hsa-miR-200b**	Downregulated	Clinical/Cell line	Tumor	GRHL2, BMI1	Pro-tumorigenic, tumor growth, EMT, metastasis, LNM, chemoresistance	(Chen et al., 2016)
**hsa-miR-200b-3p**	Downregulated	Clinical	Plasma	BMI1	Proliferation, metastasis, poor survival	(Sun et al., 2018a)
**hsa-miR-200c**	Downregulated	Clinical/Cell line	Tumor	GRHL2, BMI1, ZEB1	Pro-tumorigenic, EMT, tumor growth	(Lo et al., 2011;Kalfert et al., 2015;Chen et al., 2016)
**hsa-miR-203**	Downregulated	Clinical	Tumor	NUAK1	PI3K/Akt & p53 signaling; Invasion & EMT induction	(Manikandan et al., 2016;Obayashi et al., 2016)
**hsa-miR-204-5p**	Downregulated	Clinical/Cell line	Tumor	CXCR4, JAK2-STAT3 signaling	Proliferation, metastasis, angiogenesis, cetuximab resistance, progression	(Schneider et al., 2018)
**hsa-miR-205**	Downregulated	Clinical	Plasma		Loco-regional recurrence & poor survival	(Childs et al., 2009;Manikandan et al., 2015b)
**hsa-miR-206**	Downregulated	Clinical/Cell line	Tumor	EGFR, c-MET, K-Ras, HDAC6	Aggressiveness, proliferation, migration and invasion	(Lin et al., 2014;Koshizuka et al., 2016)
**hsa-miR-20a-5p**	Upregulated	Clinical/Cell line	Tumor			(Hui et al., 2010;Hu et al.,

						2016a;Schneider et al., 2018)
**hsa-miR-21-5p**	Upregulated	Clinical/Cell line	Tumor / Plasma/ Serum/ Blood/ Saliva/ Brush biopsy	FIH, CLU-1, DKK2 PTEN, TIMP-3, PDCD4	Tumorigenesis, Wnt/ β-catenin signaling, chemoresistance, migration, invasion, perineural invasion, poor prognosis, poor survival	(Childs et al., 2009;Hui et al., 2010;Kimura et al., 2010;Brito et al., 2014;Kawakita et al., 2014;Mydlarz et al., 2014;Kalfert et al., 2015;Manikandan et al., 2015b;Kao et al., 2016;Tseng et al., 2017;Gissi et al., 2018)
**hsa-miR-21-3p**	Upregulated	Clinical	Tumor		Cell growth, invasion, metastasis	(Tseng et al., 2017;Schneider et al., 2018)
**hsa-miR-214**	Upregulated	Clinical/Cell line	Tumor	RASSF5, FOXO3a, BMI1	Apoptosis	(Li et al., 2017)
**hsa-miR-216a**	Downregulated	Clinical/Cell line	Tumor	EIF4B	Growth & metastasis	(Li and Ma, 2015)
**hsa-miR-218**	Downregulated	Clinical/Cell line	Tumor	Laminin-332; LAMA3, LAMB3, LAMC2; Rictor (mTOR component), PPP2R5A/Wnt signaling	Invasion & migration; mTOR-Akt signaling, cisplatin resistance	(Uesugi et al., 2011;Kinoshita et al., 2012a;Zhang et al., 2017)
**hsa-miR-22**	Downregulated	Clinical / Cell line	Tumor	NLRP3	Proliferation, migration, invasion, TNM staging	(Feng et al., 2018)
**hsa-miR-220a**	Downregulated	Clinical	Saliva			(Momen-Heravi et al., 2014)
**hsa-miR-221**	Upregulated	Clinical/Cell line	Tumor	p27 & p57, MBD2, PTEN	Tumorigenesis, proliferation, invasion & metastasis	(Yang et al., 2011;He et al., 2015b)
**hsa-miR-222**	Upregulated	Clinical/Cell line	Tumor	p27 & p57, PTEN	Tumorigenesis, proliferation, invasion	(Yang et al., 2011)
**hsa-miR-222-3p**	Upregulated	Clinical	Tumor		Metastasis, clinical stage	(Chang et al., 2018;Schneider et al., 2018)
**hsa-miR-223**	Upregulated	Clinical	Tumor / Plasma / Serum		Advanced tumor stage/size	(Manikandan et al., 2016;Tachibana et al., 2016)
**hsa-miR-223-3p**	Upregulated	Clinical/ Cell line	Tumor	STAT3	Angiogenesis, cetuximab resistance	(Bozec et al., 2017)
**hsa-miR-23b**	Downregulated	Clinical	Tumor	MET	Oncogenesis & metastasis	(Fukumoto et al., 2016)
**hsa-miR-24**	Upregulated	Clinical/Cell line	Plasma / Saliva	p57	Tumor growth	(Lin et al., 2010;Momen-Heravi et al., 2014)
**hsa-miR-25**	Upregulated	Clinical	Serum			
**hsa-miR-26a**	Downregulated	Cell line	Tumor/ Saliva	TMEM184B	Migration & invasion, actin cytoskeleton pathway	(Fukumoto et al., 2015)
**hsa-miR-26b**	Downregulated	Cell line		TMEM184B	Migration & invasion, actin cytoskeleton pathway	(Fukumoto et al., 2015)
**hsa-miR-27a-3p**	Downregulated	Clinical/Cell line	Tumor	EGFR, YAP1	Tumor growth, EMT via YAP1-OCT4-Sox2 signaling	(Wu et al., 2013;Zeng et al., 2016)
**hsa-miR-27b**	Downregulated	Clinical	Tumor/ Plasma / Saliva	MET	Oncogenesis & metastasis	(Momen-Heravi et al., 2014;Fukumoto et al., 2016)
**hsa-miR-29a**	Downregulated	Clinical/Cell line	Tumor / Serum	LAMC2, ITGA6, MMP2	Invasion & metastasis	(Kinoshita et al., 2013;Manikandan et al., 2016)
**hsa-miR-29b**	Upregulated/ downregulated	Clinical/Cell line	Tumor	LAMC2, ITGA6	PI3K/Akt & p53 signaling, invasion & metastasis	(Chang et al., 2008;Kinoshita et al., 2013)
**hsa-miR-29c**	Downregulated	Clinical/Cell line	Tumor	LAMC2, ITGA7	Invasion & metastasis	(Chang et al., 2008;Kinoshita et

						al., 2013)
**hsa-miR-297**	Downregulated	Clinical/Cell line	Tumor	VEGFA	Tumor growth, poor survival	(Zhang et al., 2017)
**hsa-miR-300**	Downregulated	Cell line		Twist	EMT & metastasis	(Yu et al., 2014)
**hsa-miR-301**	Upregulated	Cell line				(Lu et al., 2012)
**hsa-miR-301-3p**	Upregulated	Clinical	Tumor			(Schneider et al., 2018)
**hsa-miR-30a-5p**	Upregulated	Cell line				(Kimura et al., 2010)
**hsa-miR-31-3p**	Upregulated	Clinical	Tumor			(Schneider et al., 2018)
**hsa-miR-31-5p**	Upregulated	Clinical /Cell line	Tumor/ Plasma/ Saliva	FIH	Tumorigenesis	(Lu et al., 2012;Hung et al., 2014;Lu et al., 2014a;Kao et al., 2016;Schneider et al., 2018)
**hsa-miR-3195**	Downregulated	Clinical	Tumor			(Schneider et al., 2018)
**hsa-miR-3196**	Downregulated	Clinical	Tumor			(Schneider et al., 2018)
**hsa-miR-32-5p**	Downregulated	Clinical	Tumor/ Serum	EZH2	Poor prognosis	(Zhang et al., 2014;Schneider et al., 2018)
**hsa-miR-320a**	Downregulated	Clinical	Tumor	Suz12	Invasion, metastasis, poor survival	(Xie et al., 2016)
**hsa-miR-323-5p**	Downregulated	Clinical	Saliva			(Momen-Heravi et al., 2014)
**hsa-miR-329**	Downregulated	Clinical	Tumor	Wnt-7b	Proliferation and invasion	(Shiah et al., 2014)
**hsa-miR-338**	Downregulated	Clinical/Cell line	Serum	NRP1	Metastasis	(Liu et al., 2015a)
**hsa-miR-340**	Downregulated	Cell line		Glut1	Tumor progression	(Xu et al., 2016)
**hsa-miR-345**	Upregulated	Clinical	Tumor		Grade dysplasia	(Brito et al., 2014)
**hsa-miR-34a**	Downregulated	Clinical/Cell line	Tumor	IL6R	p53 suppression, angiogenesis, tumor progression	(Kumar et al., 2012;Li et al., 2015b;Manikandan et al., 2015a)
**hsa-miR-363**	Downregulated	Clinical/Cell line	Tumor	PDPN, MYO1B	Invasion and metastasis, cellular migration	(Sun et al., 2013;Chapman et al., 2015)
**hsa-miR-3648**	Downregulated	Clinical	Tumor			(Schneider et al., 2018)
**hsa-miR-3651**	Downregulated	Clinical	Tumor			(Schneider et al., 2018)
**hsa-miR-3656**	Downregulated	Clinical	Tumor			(Schneider et al., 2018)
**hsa-miR-3687**	Downregulated	Clinical	Tumor			(Schneider et al., 2018)
**hsa-miR-370**	Downregulated	Clinical/Cell line	Tumor	IRS-1	Tumorigenesis	(Chang et al., 2013)
**hsa-miR-372**	Upregulated	Clinical	Plasma	P62	Nodal metastasis, lymphovascular invasion, poor survival,	(Tu et al., 2015)
**hsa-miR-373**	Upregulated	Clinical	Tumor		Nodal metastasis, lymphovascular invasion, poor survival	(Tu et al., 2015)
**hsa-miR-375**	Downregulated	Clinical/Cell line	Tumor / Plasma	IGF-IR	Cell growth, invasion through invadopodium associated extracellular matrix degradation, tumorigenesis, radio sensitivity, LNM poor survival	(Hui et al., 2010;Wiklund et al., 2011;Jimenez et al., 2015;Jung et al., 2015;Kalfert et al., 2015)
**hsa-miR-377**	Downregulated	Clinical/ Cell line	Tumor	HDAC9, NR4A1, Nur77	Growth, migration, poor survival	(Rastogi et al., 2017)
**hsa-miR-380-5p**	Downregulated	Clinical	Tumor	TP53	p53 suppression	(Manikandan et al., 2015a)
**hsa-miR-410**	Downregulated	Clinical	Tumor	Wnt-7b	Proliferation and invasion	(Shiah et al., 2014)
**hsa-miR-422a**	Downregulated	Clinical	Tumor	NT5E/CD73	Loco-regional recurrence & poor survival	(Bonnin et al., 2016)
**hsa-miR-423-5p**	Upregulated	Clinical	Tumor		Metastasis, Clinical staging	(Chang et al., 2018)

**hsa-miR-424-5p**	Upregulated	Clinical	Tumor			(Schneider et al., 2018)
**hsa-miR-429**	Downregulated	Clinical/Cell line	Tumor	ZEB1	tumor growth	(Lei et al., 2015)
**hsa-miR-433**	Downregulated	Clinical/Cell line	Tumor	HDAC6, FAK, ERK/MAPK pathway	tumor growth and metastasis, proliferation, invasion, migration, progression	(Wang et al., 2015b)
**hsa-miR-4485-3p**	Downregulated	Clinical	Tumor			(Schneider et al., 2018)
**hsa-miR-4488**	Downregulated	Clinical	Tumor			(Schneider et al., 2018)
**hsa-miR-4492**	Downregulated	Clinical	Tumor			(Schneider et al., 2018)
**hsa-miR-4497**	Downregulated	Clinical	Tumor			(Schneider et al., 2018)
**hsa-miR-4508**	Downregulated	Clinical	Tumor			(Schneider et al., 2018)
**hsa-miR-4516**	Downregulated	Clinical	Tumor			(Schneider et al., 2018)
**hsa-miR-4532**	Downregulated	Clinical	Tumor			(Schneider et al., 2018)
**hsa-miR-451**	Upregulated Downregulated	Clinical/Cell line	Tumor / Serum / Saliva	c-Myc	Relapsed, Carcinogenesis	(Hui et al., 2010;Wang et al., 2015a)
**hsa-miR-455-5p**	Upregulated	Clinical	Tumor			(Schneider et al., 2018)
**hsa-miR-483-5p**	Upregulated	Clinical	Plasma / Serum		Prognosis and lymph node metastasis	(Xu et al., 2016)
**hsa-miR-494**	Downregulated	Clinical/Cell line	Tumor	HOXA10	cell proliferation	(Chang et al., 2008;Liborio-Kimura et al., 2015)
**hsa-miR-494-3p**	Downregulated	Cell line	Tumor	BMI1	Radio sensitivity, tumor suppression, cellular senescence	(Weng et al., 2016)
**hsa-miR-497**	Upregulated	Clinical	Tumor	SMAD7	Metastasis	(Hu et al., 2016b)
**hsa-miR-499a-5p**	Downregulated	Clinical	Tumor		tumor progression	(Hou et al., 2015)
**hsa-miR-503**	Downregulated	Clinical /Cell line	Saliva			(Lu et al., 2012;Momen-Heravi et al., 2014)
**hsa-miR-506**	Downregulated	Clinical	Tumor	GATA6	Proliferation, migration, invasion, Progression	(Deng and Liu, 2015)
**hsa-miR-542-3p**	Downregulated	Clinical	Tumor	ILK, TGFβ, SMAD2/ 3	Progression, proliferation, migration, invasion	(Qiao et al., 2017)
**hsa-miR-582-5p**	Upregulated	Cell line				(Lu et al., 2012)
**hsa-miR-585**	Downregulated	Cell line	Tumor		mTOR-Akt signaling	(Uesugi et al., 2011)
**hsa-miR-6087**	Downregulated	Clinical	Tumor			(Schneider et al., 2018)
**hsa-miR-632**	Downregulated	Clinical	Saliva			(Momen-Heravi et al., 2014)
**hsa-miR-646**	Downregulated	Clinical	Saliva			(Momen-Heravi et al., 2014)
**hsa-miR-6510-3p**	Downregulated	Clinical	Tumor			(Schneider et al., 2018)
**hsa-miR-668**	Downregulated	Clinical	Saliva			(Momen-Heravi et al., 2014)
**hsa-miR-675**	Downregulated	Clinical/Cell line	Tumor		Tumorigenesis, migration, invasion, recurrence, overall survival, poor disease-free survival	(Guan et al., 2016)
**hsa-miR-7**	Upregulated	Clinical	Tumor			(Schneider et al., 2018)
**hsa-miR-708-3p**	Upregulated	Clinical	Tumor			(Schneider et al., 2018)
**hsa-miR-7704**	Downregulated	Clinical	Tumor			(Schneider et al., 2018)
**hsa-miR-874**	Downregulated	Cell line		HDAC1	cell proliferation	(Nohata et al., 2013)
**hsa-miR-877-5p**	Up/Downregulated	Clinical	Saliva			(Momen-Heravi et al., 2014;Schneider

						et al., 2018)
**hsa-miR-9**	Downregulated	Clinical/Cell line	Serum/ Saliva	CXCR4	Proliferation, tumorigenesis, poor prognosis	(Sun et al., 2016)
**hsa-miR-92a**	Upregulated	Clinical/ Cell line	Serum	FOXP1	Tumor growth, proliferation, cell cycle progression	(Guo et al., 2018)
**hsa-miR-92b**	Upregulated	Clinical/Cell line	Tumor	NLK	Tumor growth, NF-kB signaling-activation	(Liu et al., 2015b;Schneider et al., 2018)
**hsa-miR-93**	Upregulated	Clinical	Tumor		Tumor progression, metastasis & poor prognosis	(Li et al., 2015a)
**hsa-miR-944**	Upregulated	Clinical	Tumor			(Schneider et al., 2018)
**hsa-miR-98**	Downregulated	Clinical/Cell line	Tumor	IGF1R	tumor growth and metastasis	(Du et al., 2015)
**hsa-miR-99a-5p**	Downregulated	Clinical	Tumor		p53 suppression	(Manikandan et al., 2015a;Schneider et al., 2018)
**hsa-miR-99a-3p**	Downregulated	Clinical	Tumor			(Schneider et al., 2018)
**hsa-miR-99b-3p**	Downregulated	Clinical	Plasma/Saliva	GSK3β	Tumor progression	(He et al., 2015a)

References

Bonnin, N., Armandy, E., Carras, J., Ferrandon, S., Battiston-Montagne, P., Aubry, M., Guihard, S., Meyronet, D., Foy, J.P., Saintigny, P., Ledrappier, S., Jung, A., Rimokh, R., Rodriguez-Lafrasse, C., and Poncet, D. (2016). MiR-422a promotes loco-regional recurrence by targeting NT5E/CD73 in head and neck squamous cell carcinoma. *Oncotarget* 7**,** 44023-44038.

Bozec, A., Zangari, J., Butori-Pepino, M., Ilie, M., Lalvee, S., Juhel, T., Butori, C., Brest, P., Hofman, P., and Vouret-Craviari, V. (2017). MiR-223-3p inhibits angiogenesis and promotes resistance to cetuximab in head and neck squamous cell carcinoma. *Oncotarget* 8**,** 57174-57186.

Brito, J.A., Gomes, C.C., Guimaraes, A.L., Campos, K., and Gomez, R.S. (2014). Relationship between microRNA expression levels and histopathological features of dysplasia in oral leukoplakia. *J Oral Pathol Med* 43**,** 211-216.

Bufalino, A., Cervigne, N.K., De Oliveira, C.E., Fonseca, F.P., Rodrigues, P.C., Macedo, C.C., Sobral, L.M., Miguel, M.C., Lopes, M.A., Paes Leme, A.F., Lambert, D.W., Salo, T.A., Kowalski, L.P., Graner, E., and Coletta, R.D. (2015). Low miR-143/miR-145 Cluster Levels Induce Activin A Overexpression in Oral Squamous Cell Carcinomas, Which Contributes to Poor Prognosis. *PLoS One* 10**,** e0136599.

Cai, Z., Hao, X.Y., and Liu, F.X. (2018). MicroRNA-186 serves as a tumor suppressor in oral squamous cell carcinoma by negatively regulating the protein tyrosine phosphatase SHP2 expression. *Arch Oral Biol* 89**,** 20-25.

Chang, C.J., Hsu, C.C., Chang, C.H., Tsai, L.L., Chang, Y.C., Lu, S.W., Yu, C.H., Huang, H.S., Wang, J.J., Tsai, C.H., Chou, M.Y., Yu, C.C., and Hu, F.W. (2011). Let-7d functions as novel regulator of epithelial-mesenchymal transition and chemoresistant property in oral cancer. *Oncol Rep* 26**,** 1003-1010.

Chang, K.W., Chu, T.H., Gong, N.R., Chiang, W.F., Yang, C.C., Liu, C.J., Wu, C.H., and Lin, S.C. (2013). miR-370 modulates insulin receptor substrate-1 expression and inhibits the tumor phenotypes of oral carcinoma. *Oral Dis* 19**,** 611-619.

Chang, S.S., Jiang, W.W., Smith, I., Poeta, L.M., Begum, S., Glazer, C., Shan, S., Westra, W., Sidransky, D., and Califano, J.A. (2008). MicroRNA alterations in head and neck squamous cell carcinoma. *Int J Cancer* 123**,** 2791-2797.

Chang, Y.A., Weng, S.L., Yang, S.F., Chou, C.H., Huang, W.C., Tu, S.J., Chang, T.H., Huang, C.N., Jong, Y.J., and Huang, H.D. (2018). A Three-MicroRNA Signature as a Potential Biomarker for the Early Detection of Oral Cancer. *Int J Mol Sci* 19.

Chapman, B.V., Wald, A.I., Akhtar, P., Munko, A.C., Xu, J., Gibson, S.P., Grandis, J.R., Ferris, R.L., and Khan, S.A. (2015). MicroRNA-363 targets myosin 1B to reduce cellular migration in head and neck cancer. *BMC Cancer* 15**,** 861.

Chen, W., Yi, J.K., Shimane, T., Mehrazarin, S., Lin, Y.L., Shin, K.H., Kim, R.H., Park, N.H., and Kang, M.K. (2016). Grainyhead-like 2 regulates epithelial plasticity and stemness in oral cancer cells. *Carcinogenesis* 37**,** 500-510.

Childs, G., Fazzari, M., Kung, G., Kawachi, N., Brandwein-Gensler, M., Mclemore, M., Chen, Q., Burk, R.D., Smith, R.V., Prystowsky, M.B., Belbin, T.J., and Schlecht, N.F. (2009). Low-level expression of microRNAs let-7d and miR-205 are prognostic markers of head and neck squamous cell carcinoma. *Am J Pathol* 174**,** 736-745.

Datta, J., Smith, A., Lang, J.C., Islam, M., Dutt, D., Teknos, T.N., and Pan, Q. (2012). microRNA-107 functions as a candidate tumor-suppressor gene in head and neck squamous cell carcinoma by downregulation of protein kinase Cvarepsilon. *Oncogene* 31**,** 4045-4053.

Deng, L., and Liu, H. (2015). MicroRNA-506 suppresses growth and metastasis of oral squamous cell carcinoma via targeting GATA6. *Int J Clin Exp Med* 8**,** 1862-1870.

Du, Y., Li, Y., Lv, H., Zhou, S., Sun, Z., and Wang, M. (2015). miR-98 suppresses tumor cell growth and metastasis by targeting IGF1R in oral squamous cell carcinoma. *Int J Clin Exp Pathol* 8**,** 12252-12259.

Feng, X., Luo, Q., Wang, H., Zhang, H., and Chen, F. (2018). MicroRNA-22 suppresses cell proliferation, migration and invasion in oral squamous cell carcinoma by targeting NLRP3. *J Cell Physiol* 233**,** 6705-6713.

Fukumoto, I., Hanazawa, T., Kinoshita, T., Kikkawa, N., Koshizuka, K., Goto, Y., Nishikawa, R., Chiyomaru, T., Enokida, H., Nakagawa, M., Okamoto, Y., and Seki, N. (2015). MicroRNA expression signature of oral squamous cell carcinoma: functional role of microRNA-26a/b in the modulation of novel cancer pathways. *Br J Cancer* 112**,** 891-900.

Fukumoto, I., Koshizuka, K., Hanazawa, T., Kikkawa, N., Matsushita, R., Kurozumi, A., Kato, M., Okato, A., Okamoto, Y., and Seki, N. (2016). The tumor-suppressive microRNA-23b/27b cluster regulates the MET oncogene in oral squamous cell carcinoma. *Int J Oncol* 49**,** 1119-1129.

Gao, L., Ren, W., Chang, S., Guo, B., Huang, S., Li, M., Guo, Y., Li, Z., Song, T., Zhi, K., and Huang, C. (2013). Downregulation of miR-145 expression in oral squamous cell carcinomas and its clinical significance. *Onkologie* 36**,** 194-199.

Gissi, D.B., Morandi, L., Gabusi, A., Tarsitano, A., Marchetti, C., Cura, F., Palmieri, A., Montebugnoli, L., Asioli, S., Foschini, M.P., and Scapoli, L. (2018). A Noninvasive Test for MicroRNA Expression in Oral Squamous Cell Carcinoma. *Int J Mol Sci* 19.

Gombos, K., Horvath, R., Szele, E., Juhasz, K., Gocze, K., Somlai, K., Pajkos, G., Ember, I., and Olasz, L. (2013). miRNA expression profiles of oral squamous cell carcinomas. *Anticancer Res* 33**,** 1511-1517.

Gu, W.L., Ye, D.X., and Wu, J.J. (2015). [Expression and clinical significance of plasma microRNA-125b level in patients with oral squamous cell carcinoma]. *Shanghai Kou Qiang Yi Xue* 24**,** 71-75.

Guan, G.F., Zhang, D.J., Wen, L.J., Xin, D., Liu, Y., Yu, D.J., Su, K., Zhu, L., Guo, Y.Y., and Wang, K. (2016). Overexpression of lncRNA H19/miR-675 promotes tumorigenesis in head and neck squamous cell carcinoma. *Int J Med Sci* 13**,** 914-922.

Guo, J., Wen, N., Yang, S., Guan, X., and Cang, S. (2018). MiR-92a regulates oral squamous cell carcinoma (OSCC) cell growth by targeting FOXP1 expression. *Biomed Pharmacother* 104**,** 77-86.

Hauser, B., Zhao, Y., Pang, X., Ling, Z., Myers, E., Wang, P., Califano, J., and Gu, X. (2015). Functions of MiRNA-128 on the regulation of head and neck squamous cell carcinoma growth and apoptosis. *PLoS One* 10**,** e0116321.

He, B., Lin, X., Tian, F., Yu, W., and Qiao, B. (2018). MiR-133a-3p Inhibits Oral Squamous Cell Carcinoma (OSCC) Proliferation and Invasion by Suppressing COL1A1. *J Cell Biochem* 119**,** 338-346.

He, K., Tong, D., Zhang, S., Cai, D., Wang, L., Yang, Y., Gao, L., Chang, S., Guo, B., Song, T., Li, A., and Huang, C. (2015a). miRNA-99b-3p functions as a potential tumor suppressor by targeting glycogen synthase kinase-3beta in oral squamous cell carcinoma Tca-8113 cells. *Int J Oncol* 47**,** 1528-1536.

He, S., Lai, R., Chen, D., Yan, W., Zhang, Z., Liu, Z., Ding, X., and Chen, Y. (2015b). Downregulation of miR-221 Inhibits Cell Migration and Invasion through Targeting Methyl-CpG Binding Domain Protein 2 in Human Oral Squamous Cell Carcinoma Cells. *Biomed Res Int* 2015**,** 751672.

Henson, B.J., Bhattacharjee, S., O'dee, D.M., Feingold, E., and Gollin, S.M. (2009). Decreased expression of miR-125b and miR-100 in oral cancer cells contributes to malignancy. *Genes Chromosomes Cancer* 48**,** 569-582.

Hou, Y.Y., Lee, J.H., Chen, H.C., Yang, C.M., Huang, S.J., Liou, H.H., Chi, C.C., Tsai, K.W., and Ger, L.P. (2015). The association between miR-499a polymorphism and oral squamous cell carcinoma progression. *Oral Dis* 21**,** 195-206.

Hou, Y.Y., You, J.J., Yang, C.M., Pan, H.W., Chen, H.C., Lee, J.H., Lin, Y.S., Liou, H.H., Liu, P.F., Chi, C.C., Ger, L.P., and Tsai, K.W. (2016). Aberrant DNA hypomethylation of miR-196b contributes to migration and invasion of oral cancer. *Oncol Lett* 11**,** 4013-4021.

Hu, J., Ge, W., and Xu, J. (2016a). HPV 16 E7 inhibits OSCC cell proliferation, invasion, and metastasis by upregulating the expression of miR-20a. *Tumour Biol* 37**,** 9433-9440.

Hu, J., Xu, J.F., and Ge, W.L. (2016b). MiR-497 enhances metastasis of oral squamous cell carcinoma through SMAD7 suppression. *Am J Transl Res* 8**,** 3023-3031.

Hui, A.B., Lenarduzzi, M., Krushel, T., Waldron, L., Pintilie, M., Shi, W., Perez-Ordonez, B., Jurisica, I., O'sullivan, B., Waldron, J., Gullane, P., Cummings, B., and Liu, F.F. (2010). Comprehensive MicroRNA profiling for head and neck squamous cell carcinomas. *Clin Cancer Res* 16**,** 1129-1139.

Hung, P.S., Liu, C.J., Chou, C.S., Kao, S.Y., Yang, C.C., Chang, K.W., Chiu, T.H., and Lin, S.C. (2013). miR-146a enhances the oncogenicity of oral carcinoma by concomitant targeting of the IRAK1, TRAF6 and NUMB genes. *PLoS One* 8**,** e79926.

Hung, P.S., Tu, H.F., Kao, S.Y., Yang, C.C., Liu, C.J., Huang, T.Y., Chang, K.W., and Lin, S.C. (2014). miR-31 is upregulated in oral premalignant epithelium and contributes to the immortalization of normal oral keratinocytes. *Carcinogenesis* 35**,** 1162-1171.

Hunt, S., Jones, A.V., Hinsley, E.E., Whawell, S.A., and Lambert, D.W. (2011). MicroRNA-124 suppresses oral squamous cell carcinoma motility by targeting ITGB1. *FEBS Lett* 585**,** 187-192.

Islam, M., Datta, J., Lang, J.C., and Teknos, T.N. (2014). Down regulation of RhoC by microRNA-138 results in de-activation of FAK, Src and Erk1/2 signaling pathway in head and neck squamous cell carcinoma. *Oral Oncol* 50**,** 448-456.

Jimenez, L., Sharma, V.P., Condeelis, J., Harris, T., Ow, T.J., Prystowsky, M.B., Childs, G., and Segall, J.E. (2015). MicroRNA-375 Suppresses Extracellular Matrix Degradation and Invadopodial Activity in Head and Neck Squamous Cell Carcinoma. *Arch Pathol Lab Med* 139**,** 1349-1361.

Jin, Y., Chen, D., Cabay, R.J., Wang, A., Crowe, D.L., and Zhou, X. (2013). Role of microRNA-138 as a potential tumor suppressor in head and neck squamous cell carcinoma. *Int Rev Cell Mol Biol* 303**,** 357-385.

Jung, H.M., Benarroch, Y., and Chan, E.K. (2015). Anti-cancer drugs reactivate tumor suppressor miR-375 expression in tongue cancer cells. *J Cell Biochem* 116**,** 836-843.

Kalfert, D., Pesta, M., Kulda, V., Topolcan, O., Ryska, A., Celakovsky, P., Laco, J., and Ludvikova, M. (2015). MicroRNA profile in site-specific head and neck squamous cell cancer. *Anticancer Res* 35**,** 2455-2463.

Kao, S.Y., Tsai, M.M., Wu, C.H., Chen, J.J., Tseng, S.H., Lin, S.C., and Chang, K.W. (2016). Co-targeting of multiple microRNAs on factor-Inhibiting hypoxia-Inducible factor gene for the pathogenesis of head and neck carcinomas. *Head Neck* 38**,** 522-528.

Kawakita, A., Yanamoto, S., Yamada, S., Naruse, T., Takahashi, H., Kawasaki, G., and Umeda, M. (2014). MicroRNA-21 promotes oral cancer invasion via the Wnt/beta-catenin pathway by targeting DKK2. *Pathol Oncol Res* 20**,** 253-261.

Kimura, S., Naganuma, S., Susuki, D., Hirono, Y., Yamaguchi, A., Fujieda, S., Sano, K., and Itoh, H. (2010). Expression of microRNAs in squamous cell carcinoma of human head and neck and the esophagus: miR-205 and miR-21 are specific markers for HNSCC and ESCC. *Oncol Rep* 23**,** 1625-1633.

Kinoshita, T., Hanazawa, T., Nohata, N., Kikkawa, N., Enokida, H., Yoshino, H., Yamasaki, T., Hidaka, H., Nakagawa, M., Okamoto, Y., and Seki, N. (2012a). Tumor suppressive microRNA-218 inhibits cancer cell migration and invasion through targeting laminin-332 in head and neck squamous cell carcinoma. *Oncotarget* 3**,** 1386-1400.

Kinoshita, T., Nohata, N., Fuse, M., Hanazawa, T., Kikkawa, N., Fujimura, L., Watanabe-Takano, H., Yamada, Y., Yoshino, H., Enokida, H., Nakagawa, M., Okamoto, Y., and Seki, N. (2012b). Tumor suppressive microRNA-133a regulates novel targets: moesin contributes to cancer cell proliferation and invasion in head and neck squamous cell carcinoma. *Biochem Biophys Res Commun* 418**,** 378-383.

Kinoshita, T., Nohata, N., Hanazawa, T., Kikkawa, N., Yamamoto, N., Yoshino, H., Itesako, T., Enokida, H., Nakagawa, M., Okamoto, Y., and Seki, N. (2013). Tumour-suppressive microRNA-29s inhibit cancer cell migration and invasion by targeting laminin-integrin signalling in head and neck squamous cell carcinoma. *Br J Cancer* 109**,** 2636-2645.

Kong, D., Zhang, G., Ma, H., and Jiang, G. (2015). miR-1271 inhibits OSCC cell growth and metastasis by targeting ALK. *Neoplasma* 62**,** 559-566.

Koshizuka, K., Hanazawa, T., Fukumoto, I., Kikkawa, N., Matsushita, R., Mataki, H., Mizuno, K., Okamoto, Y., and Seki, N. (2016). Dual-receptor (EGFR and c-MET) inhibition by tumor-suppressive miR-1 and miR-206 in head and neck squamous cell carcinoma. *J Hum Genet*.

Koshizuka, K., Hanazawa, T., Kikkawa, N., Katada, K., Okato, A., Arai, T., Idichi, T., Osako, Y., Okamoto, Y., and Seki, N. (2018). Antitumor miR-150-5p and miR-150-3p inhibit cancer cell aggressiveness by targeting SPOCK1 in head and neck squamous cell carcinoma. *Auris Nasus Larynx* 45**,** 854-865.

Kumar, B., Yadav, A., Lang, J., Teknos, T.N., and Kumar, P. (2012). Dysregulation of microRNA-34a expression in head and neck squamous cell carcinoma promotes tumor growth and tumor angiogenesis. *PLoS One* 7**,** e37601.

Lei, W., Liu, Y.E., Zheng, Y., and Qu, L. (2015). MiR-429 inhibits oral squamous cell carcinoma growth by targeting ZEB1. *Med Sci Monit* 21**,** 383-389.

Li, G., Ren, S., Su, Z., Liu, C., Deng, T., Huang, D., Tian, Y., Qiu, Y., and Liu, Y. (2015a). Increased expression of miR-93 is associated with poor prognosis in head and neck squamous cell carcinoma. *Tumour Biol* 36**,** 3949-3956.

Li, L., and Ma, H.Q. (2015). MicroRNA-216a inhibits the growth and metastasis of oral squamous cell carcinoma by targeting eukaryotic translation initiation factor 4B. *Mol Med Rep* 12**,** 3156-3162.

Li, P., Kaslan, M., Lee, S.H., Yao, J., and Gao, Z. (2017). Progress in Exosome Isolation Techniques. *Theranostics* 7**,** 789-804.

Li, T., Li, L., Li, D., Wang, S., and Sun, J. (2015b). MiR-34a inhibits oral cancer progression partially by repression of interleukin-6-receptor. *Int J Clin Exp Pathol* 8**,** 1364-1373.

Liao, L., Wang, J., Ouyang, S., Zhang, P., Wang, J., and Zhang, M. (2015). Expression and clinical significance of microRNA-1246 in human oral squamous cell carcinoma. *Med Sci Monit* 21**,** 776-781.

Liborio-Kimura, T.N., Jung, H.M., and Chan, E.K. (2015). miR-494 represses HOXA10 expression and inhibits cell proliferation in oral cancer. *Oral Oncol* 51, 151-157.

Lin, F., Yao, L., Xiao, J., Liu, D., and Ni, Z. (2014). MiR-206 functions as a tumor suppressor and directly targets K-Ras in human oral squamous cell carcinoma. *Onco Targets Ther* 7, 1583-1591.

Lin, S.C., Kao, S.Y., Chang, J.C., Liu, Y.C., Yu, E.H., Tseng, S.H., Liu, C.J., and Chang, K.W. (2016). Up-regulation of miR-187 modulates the advances of oral carcinoma by targeting BARX2 tumor suppressor. *Oncotarget* 7, 61355-61365.

Lin, S.C., Liu, C.J., Lin, J.A., Chiang, W.F., Hung, P.S., and Chang, K.W. (2010). miR-24 up-regulation in oral carcinoma: positive association from clinical and in vitro analysis. *Oral Oncol* 46, 204-208.

Liu, C., Wang, Z., Wang, Y., and Gu, W. (2015a). MiR-338 suppresses the growth and metastasis of OSCC cells by targeting NRP1. *Mol Cell Biochem* 398, 115-122.

Liu, C.J., Lin, J.S., Cheng, H.W., Hsu, Y.H., Cheng, C.Y., and Lin, S.C. (2016). Plasma miR-187* is a potential biomarker for oral carcinoma. *Clin Oral Investig* 21, 1131-1138.

Liu, C.J., Shen, W.G., Peng, S.Y., Cheng, H.W., Kao, S.Y., Lin, S.C., and Chang, K.W. (2014). miR-134 induces oncogenicity and metastasis in head and neck carcinoma through targeting WWOX gene. *Int J Cancer* 134, 811-821.

Liu, C.J., Tsai, M.M., Tu, H.F., Lui, M.T., Cheng, H.W., and Lin, S.C. (2013). miR-196a overexpression and miR-196a2 gene polymorphism are prognostic predictors of oral carcinomas. *Ann Surg Oncol* 20 Suppl 3, S406-414.

Liu, X., Jiang, L., Wang, A., Yu, J., Shi, F., and Zhou, X. (2009). MicroRNA-138 suppresses invasion and promotes apoptosis in head and neck squamous cell carcinoma cell lines. *Cancer Lett* 286, 217-222.

Liu, Z., Diep, C., Mao, T., Huang, L., Merrill, R., Zhang, Z., and Peng, Y. (2015b). MicroRNA-92b promotes tumor growth and activation of NF-kappaB signaling via regulation of NLK in oral squamous cell carcinoma. *Oncol Rep* 34, 2961-2968.

Lo, W.L., Yu, C.C., Chiou, G.Y., Chen, Y.W., Huang, P.I., Chien, C.S., Tseng, L.M., Chu, P.Y., Lu, K.H., Chang, K.W., Kao, S.Y., and Chiou, S.H. (2011). MicroRNA-200c attenuates tumour growth and metastasis of presumptive head and neck squamous cell carcinoma stem cells. *J Pathol* 223, 482-495.

Lu, W.C., Kao, S.Y., Yang, C.C., Tu, H.F., Wu, C.H., Chang, K.W., and Lin, S.C. (2014a). EGF up-regulates miR-31 through the C/EBPbeta signal cascade in oral carcinoma. *PLoS One* 9, e108049.

Lu, Y.C., Chang, J.T., Liao, C.T., Kang, C.J., Huang, S.F., Chen, I.H., Huang, C.C., Huang, Y.C., Chen, W.H., Tsai, C.Y., Wang, H.M., Yen, T.C., You, G.R., Chiang, C.H., and Cheng, A.J. (2014b). OncomiR-196 promotes an invasive phenotype in oral cancer through the NME4-JNK-TIMP1-MMP signaling pathway. *Mol Cancer* 13, 218.

Lu, Y.C., Chen, Y.J., Wang, H.M., Tsai, C.Y., Chen, W.H., Huang, Y.C., Fan, K.H., Tsai, C.N., Huang, S.F., Kang, C.J., Chang, J.T., and Cheng, A.J. (2012). Oncogenic function and early detection potential of miRNA-10b in oral cancer as identified by microRNA profiling. *Cancer Prev Res (Phila)* 5, 665-674.

Manikandan, M., Deva Magendhra Rao, A.K., Arunkumar, G., Manickavasagam, M., Rajkumar, K.S., Rajaraman, R., and Munirajan, A.K. (2016). Oral squamous cell carcinoma: microRNA expression profiling and integrative analyses for elucidation of tumourigenesis mechanism. *Mol Cancer* 15, 28.

Manikandan, M., Deva Magendhra Rao, A.K., Arunkumar, G., Rajkumar, K.S., Rajaraman, R., and Munirajan, A.K. (2015a). Down Regulation of miR-34a and miR-143 May Indirectly Inhibit p53 in Oral Squamous Cell Carcinoma: a Pilot Study. *Asian Pac J Cancer Prev* 16, 7619-7625.

Manikandan, M., Deva Magendhra Rao, A.K., Rajkumar, K.S., Rajaraman, R., and Munirajan, A.K. (2015b). Altered levels of miR-21, miR-125b-2*, miR-138, miR-155, miR-184, and miR-205 in oral squamous cell carcinoma and association with clinicopathological characteristics. *J Oral Pathol Med* 44, 792-800.

Momen-Heravi, F., Trachtenberg, A.J., Kuo, W.P., and Cheng, Y.S. (2014). Genomewide Study of Salivary MicroRNAs for Detection of Oral Cancer. *J Dent Res* 93**,** 86S-93S.

Mydlarz, W., Uemura, M., Ahn, S., Hennessey, P., Chang, S., Demokan, S., Sun, W., Shao, C., Bishop, J., Krosting, J., Mambo, E., Westra, W., Ha, P., Sidransky, D., and Califano, J. (2014). Clusterin is a gene-specific target of microRNA-21 in head and neck squamous cell carcinoma. *Clin Cancer Res* 20**,** 868-877.

Ni, Y.H., Huang, X.F., Wang, Z.Y., Han, W., Deng, R.Z., Mou, Y.B., Ding, L., Hou, Y.Y., and Hu, Q.G. (2014). Upregulation of a potential prognostic biomarker, miR-155, enhances cell proliferation in patients with oral squamous cell carcinoma. *Oral Surg Oral Med Oral Pathol Oral Radiol* 117**,** 227-233.

Nohata, N., Hanazawa, T., Kinoshita, T., Inamine, A., Kikkawa, N., Itesako, T., Yoshino, H., Enokida, H., Nakagawa, M., Okamoto, Y., and Seki, N. (2013). Tumour-suppressive microRNA-874 contributes to cell proliferation through targeting of histone deacetylase 1 in head and neck squamous cell carcinoma. *Br J Cancer* 108**,** 1648-1658.

Nohata, N., Sone, Y., Hanazawa, T., Fuse, M., Kikkawa, N., Yoshino, H., Chiyomaru, T., Kawakami, K., Enokida, H., Nakagawa, M., Shozu, M., Okamoto, Y., and Seki, N. (2011). miR-1 as a tumor suppressive microRNA targeting TAGLN2 in head and neck squamous cell carcinoma. *Oncotarget* 2**,** 29-42.

Obayashi, M., Yoshida, M., Tsunematsu, T., Ogawa, I., Sasahira, T., Kuniyasu, H., Imoto, I., Abiko, Y., Xu, D., Fukunaga, S., Tahara, H., Kudo, Y., Nagao, T., and Takata, T. (2016). microRNA-203 suppresses invasion and epithelial-mesenchymal transition induction via targeting NUAK1 in head and neck cancer. *Oncotarget* 7**,** 8223-8239.

Piao, L., Zhang, M., Datta, J., Xie, X., Su, T., Li, H., Teknos, T.N., and Pan, Q. (2012). Lipid-based nanoparticle delivery of Pre-miR-107 inhibits the tumorigenicity of head and neck squamous cell carcinoma. *Mol Ther* 20**,** 1261-1269.

Qiao, B., Cai, J.H., King-Yin Lam, A., and He, B.X. (2017). MicroRNA-542-3p inhibits oral squamous cell carcinoma progression by inhibiting ILK/TGF-beta1/Smad2/3 signaling. *Oncotarget* 8**,** 70761-70776.

Rastogi, B., Kumar, A., Raut, S.K., Panda, N.K., Rattan, V., Joshi, N., and Khullar, M. (2017). Downregulation of miR-377 Promotes Oral Squamous Cell Carcinoma Growth and Migration by Targeting HDAC9. *Cancer Invest* 35**,** 152-162.

Sasahira, T., Kurihara, M., Bhawal, U.K., Ueda, N., Shimomoto, T., Yamamoto, K., Kirita, T., and Kuniyasu, H. (2012). Downregulation of miR-126 induces angiogenesis and lymphangiogenesis by activation of VEGF-A in oral cancer. *Br J Cancer* 107**,** 700-706.

Schneider, A., Victoria, B., Lopez, Y.N., Suchorska, W., Barczak, W., Sobecka, A., Golusinski, W., Masternak, M.M., and Golusinski, P. (2018). Tissue and serum microRNA profile of oral squamous cell carcinoma patients. *Sci Rep* 8**,** 675.

Shao, Y., Qu, Y., Dang, S., Yao, B., and Ji, M. (2013). MiR-145 inhibits oral squamous cell carcinoma (OSCC) cell growth by targeting c-Myc and Cdk6. *Cancer Cell Int* 13**,** 51.

Shi, L.J., Zhang, C.Y., Zhou, Z.T., Ma, J.Y., Liu, Y., Bao, Z.X., and Jiang, W.W. (2015). MicroRNA-155 in oral squamous cell carcinoma: Overexpression, localization, and prognostic potential. *Head Neck* 37**,** 970-976.

Shiah, S.G., Hsiao, J.R., Chang, W.M., Chen, Y.W., Jin, Y.T., Wong, T.Y., Huang, J.S., Tsai, S.T., Hsu, Y.M., Chou, S.T., Yen, Y.C., Jiang, S.S., Shieh, Y.S., Chang, I.S., Hsiao, M., and Chang, J.Y. (2014). Downregulated miR329 and miR410 promote the proliferation and invasion of oral squamous cell carcinoma by targeting Wnt-7b. *Cancer Res* 74**,** 7560-7572.

Shin, K.H., Bae, S.D., Hong, H.S., Kim, R.H., Kang, M.K., and Park, N.H. (2011). miR-181a shows tumor suppressive effect against oral squamous cell carcinoma cells by downregulating K-ras. *Biochem Biophys Res Commun* 404**,** 896-902.

Suh, Y.E., Raulf, N., Gaken, J., Lawler, K., Urbano, T.G., Bullenkamp, J., Gobeil, S., Huot, J., Odell, E., and Tavassoli, M. (2015). MicroRNA-196a promotes an oncogenic effect in head and neck

cancer cells by suppressing annexin A1 and enhancing radioresistance. *Int J Cancer* 137, 1021-1034.

Sun, G., Cao, Y., Wang, P., Song, H., Bie, T., Li, M., and Huai (2018a). miR-200b-3p in plasma is a potential diagnostic biomarker in oral squamous cell carcinoma. *Biomarkers* 23, 137-141.

Sun, L., Liu, L., Fu, H., Wang, Q., and Shi, Y. (2016). Association of Decreased Expression of Serum miR-9 with Poor Prognosis of Oral Squamous Cell Carcinoma Patients. *Med Sci Monit* 22, 289-294.

Sun, Q., Zhang, J., Cao, W., Wang, X., Xu, Q., Yan, M., Wu, X., and Chen, W. (2013). Dysregulated miR-363 affects head and neck cancer invasion and metastasis by targeting podoplanin. *Int J Biochem Cell Biol* 45, 513-520.

Sun, Y., Hu, B., Wang, Q., Ye, M., Qiu, Q., Zhou, Y., Zeng, F., Zhang, X., Guo, Y., and Guo, L. (2018b). Long non-coding RNA HOTTIP promotes BCL-2 expression and induces chemoresistance in small cell lung cancer by sponging miR-216a. *Cell Death Dis* 9, 85.

Tachibana, H., Sho, R., Takeda, Y., Zhang, X., Yoshida, Y., Narimatsu, H., Otani, K., Ishikawa, S., Fukao, A., Asao, H., and Iino, M. (2016). Circulating miR-223 in Oral Cancer: Its Potential as a Novel Diagnostic Biomarker and Therapeutic Target. *PLoS One* 11, e0159693.

Tseng, H.H., Tseng, Y.K., You, J.J., Kang, B.H., Wang, T.H., Yang, C.M., Chen, H.C., Liou, H.H., Liu, P.F., Ger, L.P., and Tsai, K.W. (2017). Next-generation Sequencing for microRNA Profiling: MicroRNA-21-3p Promotes Oral Cancer Metastasis. *Anticancer Res* 37, 1059-1066.

Tu, H.F., Chang, K.W., Cheng, H.W., and Liu, C.J. (2015). Upregulation of miR-372 and -373 associates with lymph node metastasis and poor prognosis of oral carcinomas. *Laryngoscope* 125, E365-370.

Tu, H.F., Liu, C.J., Chang, C.L., Wang, P.W., Kao, S.Y., Yang, C.C., Yu, E.H., Lin, S.C., and Chang, K.W. (2012). The association between genetic polymorphism and the processing efficiency of miR-149 affects the prognosis of patients with head and neck squamous cell carcinoma. *PLoS One* 7, e51606.

Uesugi, A., Kozaki, K., Tsuruta, T., Furuta, M., Morita, K., Imoto, I., Omura, K., and Inazawa, J. (2011). The tumor suppressive microRNA miR-218 targets the mTOR component Rictor and inhibits AKT phosphorylation in oral cancer. *Cancer Res* 71, 5765-5778.

Wang, H., Zhang, G., Wu, Z., Lu, B., Yuan, D., Li, X., and Lu, Z. (2015a). MicoRNA-451 is a novel tumor suppressor via targeting c-myc in head and neck squamous cell carcinomas. *J Cancer Res Ther* 11 Suppl 2, C216-221.

Wang, L., and Liu, H. (2016). microRNA-188 is downregulated in oral squamous cell carcinoma and inhibits proliferation and invasion by targeting SIX1. *Tumour Biol* 37, 4105-4113.

Wang, T., Ren, Y., Liu, R., Ma, J., Shi, Y., Zhang, L., and Bu, R. (2017). miR-195-5p Suppresses the Proliferation, Migration, and Invasion of Oral Squamous Cell Carcinoma by Targeting TRIM14. *Biomed Res Int* 2017, 7378148.

Wang, X.C., Ma, Y., Meng, P.S., Han, J.L., Yu, H.Y., and Bi, L.J. (2015b). miR-433 inhibits oral squamous cell carcinoma (OSCC) cell growth and metastasis by targeting HDAC6. *Oral Oncol* 51, 674-682.

Weng, J.H., Yu, C.C., Lee, Y.C., Lin, C.W., Chang, W.W., and Kuo, Y.L. (2016). miR-494-3p Induces Cellular Senescence and Enhances Radiosensitivity in Human Oral Squamous Carcinoma Cells. *Int J Mol Sci* 17.

Wiklund, E.D., Gao, S., Hulf, T., Sibbritt, T., Nair, S., Costea, D.E., Villadsen, S.B., Bakholdt, V., Bramsen, J.B., Sorensen, J.A., Krogdahl, A., Clark, S.J., and Kjems, J. (2011). MicroRNA alterations and associated aberrant DNA methylation patterns across multiple sample types in oral squamous cell carcinoma. *PLoS One* 6, e27840.

Wu, X., Bhayani, M.K., Dodge, C.T., Nicoloso, M.S., Chen, Y., Yan, X., Adachi, M., Thomas, L., Galer, C.E., Jiffar, T., Pickering, C.R., Kupferman, M.E., Myers, J.N., Calin, G.A., and Lai, S.Y. (2013). Coordinated targeting of the EGFR signaling axis by microRNA-27a*. *Oncotarget* 4, 1388-1398.

Xie, N., Wang, C., Zhuang, Z., Hou, J., Liu, X., Wu, Y., Liu, H., and Huang, H. (2016). Decreased miR-320a promotes invasion and metastasis of tumor budding cells in tongue squamous cell carcinoma. *Oncotarget* 7**,** 65744-65757.

Xu, H., Yang, Y., Zhao, H., Yang, X., Luo, Y., Ren, Y., Liu, W., and Li, N. (2016). Serum miR-483-5p: a novel diagnostic and prognostic biomarker for patients with oral squamous cell carcinoma. *Tumour Biol* 37**,** 447-453.

Xu, R., Zeng, G., Gao, J., Ren, Y., Zhang, Z., Zhang, Q., Zhao, J., Tao, H., and Li, D. (2015). miR-138 suppresses the proliferation of oral squamous cell carcinoma cells by targeting Yes-associated protein 1. *Oncol Rep* 34**,** 2171-2178.

Yang, C.J., Shen, W.G., Liu, C.J., Chen, Y.W., Lu, H.H., Tsai, M.M., and Lin, S.C. (2011). miR-221 and miR-222 expression increased the growth and tumorigenesis of oral carcinoma cells. *J Oral Pathol Med* 40**,** 560-566.

Yang, X., Wu, H., and Ling, T. (2014). Suppressive effect of microRNA-126 on oral squamous cell carcinoma in vitro. *Mol Med Rep* 10**,** 125-130.

Yu, J., Xie, F., Bao, X., Chen, W., and Xu, Q. (2014). miR-300 inhibits epithelial to mesenchymal transition and metastasis by targeting Twist in human epithelial cancer. *Mol Cancer* 13**,** 121.

Zeng, G., Xun, W., Wei, K., Yang, Y., and Shen, H. (2016). MicroRNA-27a-3p regulates epithelial to mesenchymal transition via targeting YAP1 in oral squamous cell carcinoma cells. *Oncol Rep* 36**,** 1475-1482.

Zhang, B., Li, Y., Hou, D., Shi, Q., Yang, S., and Li, Q. (2017). MicroRNA-375 Inhibits Growth and Enhances Radiosensitivity in Oral Squamous Cell Carcinoma by Targeting Insulin Like Growth Factor 1 Receptor. *Cell Physiol Biochem* 42**,** 2105-2117.

Zhang, D., Ni, Z., Xu, X., and Xiao, J. (2014). MiR-32 functions as a tumor suppressor and directly targets EZH2 in human oral squamous cell carcinoma. *Med Sci Monit* 20**,** 2527-2535.

Zhuang, Z., Xie, N., Hu, J., Yu, P., Wang, C., Hu, X., Han, X., Hou, J., Huang, H., and Liu, X. (2017). Interplay between DeltaNp63 and miR-138-5p regulates growth, metastasis and stemness of oral squamous cell carcinoma. *Oncotarget* 8**,** 21954-21973.
